# NRF2 activation ameliorates blood–brain barrier injury after cerebral ischemic stroke by regulating ferroptosis and inflammation

**DOI:** 10.1038/s41598-024-53836-0

**Published:** 2024-03-04

**Authors:** Wei Fan, Hongping Chen, Meng Li, Xuehui Fan, Fangchao Jiang, Chen Xu, Yingju Wang, Wan Wei, Jihe Song, Di Zhong, Guozhong Li

**Affiliations:** 1 Department of Neurology, The First Affiliated Hospital of Harbin Medical University, 23 You Zheng Street, Harbin, 150001 Heilongjiang Province People’s Republic of China; 2 Department of Neurology, Heilongjiang Provincial Hospital, 82 Zhong Shan Street, Harbin, 150001 Heilongjiang Province People’s Republic of China

**Keywords:** Ischemic stroke, NRF2, Blood–brain barrier, Inflammation, Ferroptosis, Molecular medicine, Neurology

## Abstract

Arterial occlusion-induced ischemic stroke (IS) is a highly frequent stroke subtype. Nuclear factor erythroid 2-related factor 2 (NRF2) is a transcription factor that modulates antioxidant genes. Its role in IS is still unelucidated. The current study focused on constructing a transient middle cerebral artery occlusion (tMCAO) model for investigating the NRF2-related mechanism underlying cerebral ischemia/reperfusion (I/R) injury. Each male C57BL/6 mouse was injected with/with no specific NRF2 activator post-tMCAO. Changes in blood–brain barrier (BBB)-associated molecule levels were analyzed using western-blotting, PCR, immunohistochemistry, and immunofluorescence analysis. NRF2 levels within cerebral I/R model decreased at 24-h post-ischemia. NRF2 activation improved brain edema, infarct volume, and neurological deficits after MCAO/R. Similarly, sulforaphane (SFN) prevented the down-regulated tight junction proteins occludin and zonula occludens 1 (ZO-1) and reduced the up-regulated aquaporin 4 (AQP4) and matrix metalloproteinase 9 (MMP9) after tMCAO. Collectively, NRF2 exerted a critical effect on preserving BBB integrity modulating ferroptosis and inflammation. Because NRF2 is related to BBB injury regulation following cerebral I/R, this provides a potential therapeutic target and throws light on the underlying mechanism for clinically treating IS.

## Introduction

Globally, stroke has the second highest mortality rate and is third among the causes of disability-adjusted life-years (DALYs)^1^. Stroke poses the greatest health expenditure burdens among low-to-middle-income countries^2^. Ischemic stroke (IS) occupies ~ 80% of stroke^3^. IS may occur because of cerebral ischemia due to cerebral vascular thrombosis. The main pathophysiological characteristic of IS blood–brain barrier (BBB) injury that induces severe clinical complications. BBB primarily comprises brain microvascular endothelial cells (BMECs), and these cells express intercellular junctions, mainly including adherent junction (AJ) and tight junction (TJ)^4^. The BBB refers to the dynamic part of brain-vascular interface for maintaining brain homeostasis and regulating penetration of solutes in the brain tissue^5^. In the case of cerebral ischemia, BBB is injured, which then induces increased vascular permeability, leukocyte infiltration, eventually resulting in brain edema and worsening cerebral infarction during IS. BBB disruption promotes secondary brain injury while enhancing the hemorrhage transformation incidence, thereby severely affecting the IS prognosis^6^. However, the underlying molecular mechanisms of BBB injury in IS are not entirely elucidated.

Nuclear factor erythroid 2-related factor 2 (NRF2), the transcription factor, and basic leucine zipper (bZIP) transcription factors can maintain the low expression by degradation and ubiquitination through combination with kelch-like ECH-associated protein 1 (Keap1), the corresponding negative regulatory molecule^7^. The elevated oxidative stress level in cells enhances NRF2 dissociation and nuclear translocation; thereafter, NRF2 can interact with the antioxidant response elements (AREs) within target gene promoter, eventually up-regulating its transcription. NRF2 binds to small MAF proteins, modulates numerous antioxidant genes, and affects the endogenous antioxidant defense system^8–10^. Excessive reactive oxygen species (ROS) production is a primary mechanism explaining BBB disruption^11^. In the brain microvascular system, the activation of NRF2 defense pathway is beneficial for preventing BBB disruption and neurological impairment during IS^12^. While NRF2 deficiency can increase the intracellular ROS content, suggesting that it is important for maintaining ROS homeostasis^13^. Released NRF2 triggers antioxidant protein-encoding gene transcription, including superoxide dismutase (SOD), catalase (CAT), heme oxygenase 1 (HO-1), and NAD(P)H: quinone oxidoreductase 1 (NQO1)^14^. In addition, NRF2 modulates tissue repair- and remodeling-related and anti-inflammatory genes, suggesting that it has functions other than antioxidation and detoxification^15^. When NRF2/HO-1 is activated within the cerebral microvessels in the peri-infarct area, potent antioxidant effects are observed; consequently, the infarction volume decreases, and the BBB injury and neurological deficit in IS mice are alleviated^16^. Ferroptosis is the novel regulatory cell death resulting from the excess production of iron-dependent reactive oxygen species and lipid peroxides^17^. Ferroptosis inhibitors ferrostatin-1 or liproxstatin-1 prevent neuronal damage in tMCAO mice models^18,19^. We studied the impact of ferroptosis on BBB injury, summarized the progress in the ferroptosis-related mechanism, and discussed how diverse ferroptosis-related factors affect the severity of BBB injury and prognostic outcome. The results of the present study offer superior therapeutic targets for IS. NRF2 regulates the key ferroptosis proteins, including the light and heavy chains of ferritin (FTL/FTH1), ferroportin (FPN1), SLC7A11, glutathione (GSH), and glutathione peroxidase 4 (GPX4), to inhibit iron overload and lipid peroxidation (LPO)^20–22^. The upregulation of NRF2 expression would reduce inflammation and enhance the anti-inflammatory effect^23,24^. Actually, NRF2 activation can prevent BBB disruption during cerebral ischemia/reperfusion (I/R) injury^25^.

Herein, we used the NRF2 activator sulforaphane (SFN), investigated the neurological deficits and infarct volume of mice in I/R injury, and eventually studied the protective effect of NRF2 in cerebral I/R damage and the associated molecular mechanism in disrupted BBB structure.

## Materials and methods

### Animal experiments

The SPF-grade C57BL/6 male mice weighing ~ 20–25 g were provided by Liaoning Changsheng Biotechnology and treated strictly according to the Laboratory Animal Care guidelines from the First Affiliated Hospital of Harbin Medical University, based on the recommendations of the US National Institutes of Health and the ARRIVE guidelines (https://arriveguidelines.org). All animals were raised at 23–25 °C, 50–60% humidity, as well as 12-h/12-h light/dark cycle conditions. They freely consumed food and water.

### Transient middle cerebral artery occlusion model (tMCAO) establishment

After giving isoflurane (2–3% oxygen) for anesthesia, each mouse was placed in the supine position^26^. A homoeothermic blanket was employed to keep a rectal temperature of 37 °C intraoperatively. Thereafter, an incision was made in the cervical midline, followed by the separation of the common carotid artery (CCA), external carotid artery (ECA), together with internal carotid artery (ICA) in succession. Then, occlusion of the middle cerebral artery (MCA) origin was completed with the Doccol suture (diameter, 0.21 mm) from the ECA incision along the bifurcation of ICA and ECA at a depth of ~ 9 ± 1 mm^27^. The thread was terminated in the case of resistance. After suture removal 1 h later, the mice recovered for 6 h, 24 h, 3 d, 5 d, and 7 d^28^. Sham mice received an identical procedure without inserting the intraluminal filament. One technician was responsible for performing all operations. Sulforaphane (SFN, HY-13755, MCE) was dissolved within dimethylsulfoxide (DMSO, < 2%). For the SFN + tMCAO and V + tMCAO groups, each mouse was given an intraperitoneal injection of SFN at 5 mg/kg or vehicle (DMSO < 2%) during reperfusion^16^. Thereafter, the animals were divided into the (1) sham, (2) tMCAO, (3) SFN + tMCAO, and (4) V + tMCAO groups.

### Neurological deficit evaluation

Neurological deficits were used for assessing the IS severity. At 24-h post-ischemia, neurological deficits were scored in a blinded manner, with 0–5: 0 indicating the absence of neurological deficits; 1, forelimb weakness and torso turning on ipsilateral side in the case of tail suspension; 2, the inability of left forelimb extension; 3, circling on the left side; 4, falling on the contralateral side; and 5, low consciousness level, absence of spontaneous activity or death.

### Brain water content determination

The wet/dry approach was adopted for measuring brain water content for evaluating brain edema, as described in a previous report^29^. After sacrifice, the right side of the brain was collected at once. Thereafter, brain weight was measured as the wet weight. The samples were further baked using a 100 °C oven till the weight remained unchanged. Brain water content (%) = (wet weight − dry weight)/wet weight × 100%.

### Evans blue extravasation

With the purpose of analyzing BBB permeability changes, the mice were exposed to intraperitoneal injection with 2% Evans blue solution at 4 mL/kg. Following reperfusion for 24 h, normal saline was added for heart perfusion. Then, the dissected brain tissues were subjected to homogenization and centrifugation to collect supernatants for measurements using a spectrophotometer at 620 nm.

### Infarct area determination

Mice were sacrificed to collect brain tissues, which were later prepared in seven sheets (1-mm in thickness) from the anterior top of the frontal lobe. Thereafter, sheets were incubated with 2% 2,3,5-triphenyltetrazolium chloride solution (TTC, Solarbio) in dark under 37 °C for half an hour, followed by fixation with 4% paraformaldehyde. Later, a camera was used to acquire photographs of the brain sections, and ImageJ software was used for determining the infarction volume as follows: infarction volume (%) = (contralateral hemisphere volume − ipsilateral hemisphere non-infarct volume)/contralateral hemisphere volume × 100%.

### Western blot (WB) analysis

Right brain tissue was homogenized with the use of RIPA lysis buffer (Beyotime, China) which involved protease and phosphatase inhibitors (Beyotime, China). Then, the tissues were subjected to centrifugation at 12,000×*g* for 15 min. The BCA protein detection kit (Beyotime, China) was adopted for measuring protein contents. Thereafter, 30 µg proteins were loaded for separation using 10% or 12.5% SDS-PAGE before electrotransfer on polyvinylidene difluoride (PVDF) filters. Thereafter, nonfat 5% milk was added to block the membranes for 1 h. The membranes were cut prior to hybridization with antibodies. Then the membrances were followed by overnight incubation at 4 °C using primary antibodies: anti-NRF2 (1:1000, ab62352, Abcam), anti-Occludin (1:1000, 27260-AP, Proteintech), anti-AQP4 (1:1000, 16473-1-AP, Proteintech), anti-MMP9 (1:1000, 16375-1-AP, Proteintech), anti-ZO-1 (1:1000, 21773-1-AP, Proteintech), anti-ACSL4 (1:2000, 22401-1-AP, Proteintech), anti-XCT(1:2000, 26864-1-AP, Proteintech), anti-GPX4 (1:3000, 67763-1-Ig, Proteintech), anti-GAPDH (1:50000, 60004-1-Ig, Proteintech), anti-p-NF-κB (1:1000, AF2006, Affinity), anti-IL-18 (1:1000, 10663-1-AP, Proteintech), and anti-IL-1β (1:1000, AF4006, Affinity). After washing by TBST, membranes were incubated using horseradish peroxidase-labeled secondary antibodies for 60 min. In addition, an enhanced chemiluminescence detection kit was adopted for protein signal visualization. ImageJ was used for signal quantification, with GAPDH as the reference. The western blot original images are provided as Supplementary Fig. S1 available with this article online.

### Quantitative real-time PCR

We adopted Trizol reagent (Invitrogen, Thermo Fisher Scientific) for extracting total brain tissue RNA, which was further prepared reverse transcribed to cDNA using reverse transcriptase ((TIANGEN, China) in line with specific protocols. Quantitative PCR was conducted with the use of a SYBR green kit (TIANGEN, China) and a PCR machine instrument (BioRad, Singapore). The sequences of the primers used are shown below: AQP4, forward (F): CTTTCTGGAAGGCAGTCTCAG, reverse (R): CCACACCGAGCAAAACAAAGAT; MMP9, F: CTGGACAGCCAGACACTAAAG, R:CTCGCGGCAAGTCTTCAGAG; ZO-1, F: GCCGCTAAGAGCACAGCAA, R: TCCCCACTCTGAAAATGAGGA; Occludin, F: TTGAAAGTCCACCTCCTTACAGA, R: CCGGATAAAAAGAGTACGCTGG; and GAPDH, F: AGGTCGGTGTGAACGGATTTG, R: TGTAGACCATGTAGTTGAGGTCA. Finally, the gene level was analyzed using the 2^–∆∆Ct^ approach.

### Immunohistochemistry

After dewaxing, paraffin-embedded sections were rehydrated, and subject to incubation using 0.3% hydrogen peroxide. Thereafter, the sections were heated with a citric acid solution for 4 min within the pressure cooker to achieve antigen retrieval. After 30-min blocking using 5% BSA, the 5-µm sections were subjected to overnight incubation with primary antibodies (Proteintech), including anti-ZO-1 (1:50, 21773-1-AP), anti-AQP4 (1:100, 16473-1-AP), and anti-MMP9 (1:50, 16375-1-AP) under 4 °C, before another 30-min incubation with the secondary antibody (PV-6002; Zhongshan Biotechnology) at 37 °C. Finally, diaminobenzidine (DAB, ZLI-9018; Zhongshan Biotechnology) was added to develop color, and the microscope was employed for section observation. Integrated optical density (IOD) values were explored with Image-Pro Plus 6.0.

### Double-immunofluorescence analysis

The 4% paraformaldehyde was added to fix brain sections before 20-min permeabilization using 0.2% Triton X-100. Later, primary antibodies were added to incubate sections overnight under 4 °C, including anti-CD31 (Santa Cruz, sc-376764, 1:100), anti-ZO-1 (Abmart, TA5145, 1:100), anti-occludin (Wanlei, WL01996, 1:50), anti-MMP9 (Wanlei, WL03096, 1:50), and anti-AQP4 (Proteintech, 16473-1-AP, 1:100) antibodies, the 7-µm sections were further incubated with suitable secondary antibodies (BOSTER, BA1089, and BA1105, 1:100) in dark under ambient temperature for 3 h. Cell nuclei were stained using DAPI (Abcam, ab104139). In addition, the fluorescence microscope (Nikon, Y-TV55, JAPAN) was adopted for imaging, while ImageJ was used for analyzing immunofluorescence. This study selected the regions surrounding ischemia in the ischemic hemisphere for imaging. This assay was double-blinded, and different researchers were responsible for image reading and statistical analysis.

### Iron and glutathione (GSH) level detection

Ischemic brain tissues were homogenized. The supernatants were harvested to determine the iron and GSH contents. Every operation was performed using the Iron (Cat#A039–2–1) and Reduced Glutathione (Cat#A006) Detection Kits (Nanjing Jiancheng Bioengineering Institute, China) in line with specific guidance.

### Lipid peroxidation (LPO) analysis

Brain tissue was prepared following the aforementioned description. Thereafter, at 24-h post-MCAO, the LPO level within brain tissue lysates surrounding infarct area was identified based on the LPO kit (Nanjing Jiancheng Bioengineering Institute Cat#A106–1, China), in line with the manufacturer’s protocol. Data were expressed as micromoles/gram total protein (µmol/g prot). Then, with the purpose of measuring protein content, we used BCA protein assay reagent kit (Beyotime, China).

### Measurement of malondialdehyde and superoxide dismutase levels

Following the manufacturer’s protocols, we assessed malondialdehyde (MDA) and superoxide dismutase (SOD) levels with the lipid peroxidation MDA assay kit (Beyotime, China, S0131S) as well as total SOD assay kit (Beyotime, China, S0109).

### Reactive oxygen species production

ROS level was measured. DCFH-DA Dye was diluted using DMSO and loading buffer solution till 10 µmol/L. Thereafter, DCFH-DA was supplemented to incubate tissues at 37 °C for 3 h. In addition, to analyze fluorescence intensity, a fluorescence microscope was employed.

### Transmission electron microscopy

Transmission electron microscopy (TEM, Japan Electron microscopy Hitachi, H-7650) was performed to analyze ultrastructure in the peri-infarct area 24 h post-ischemia. First, 4% paraformaldehyde solution was added for mouse perfusion. Second, the tissue was prepared in 1 mm^3^ cubes and steeped within 2.5% glutaraldehyde and 1% osmic acid. After a series of operations, including dehydration, embedding, and slicing, lead citrate and uranyl acetate were added for section staining. Fields from every sample were randomly viewed to calculate the ferroptotic mitochondrial number.

### Statistical analysis

GraphPad Prism software 8.0 was employed to perform statistical analysis. Results were shown to be means ± SEM. One-way ANOVA and Tukey post hoc test were adopted for analyzing differences between the two groups. *p* < 0.05 implied statistical significance.

### Ethics approval

The animal study was reviewed and approved by the Committee of the First Affiliated Hospital of Harbin Medical University.

## Results

### NRF2 is downregulated following tMCAO

**Figure 1:**
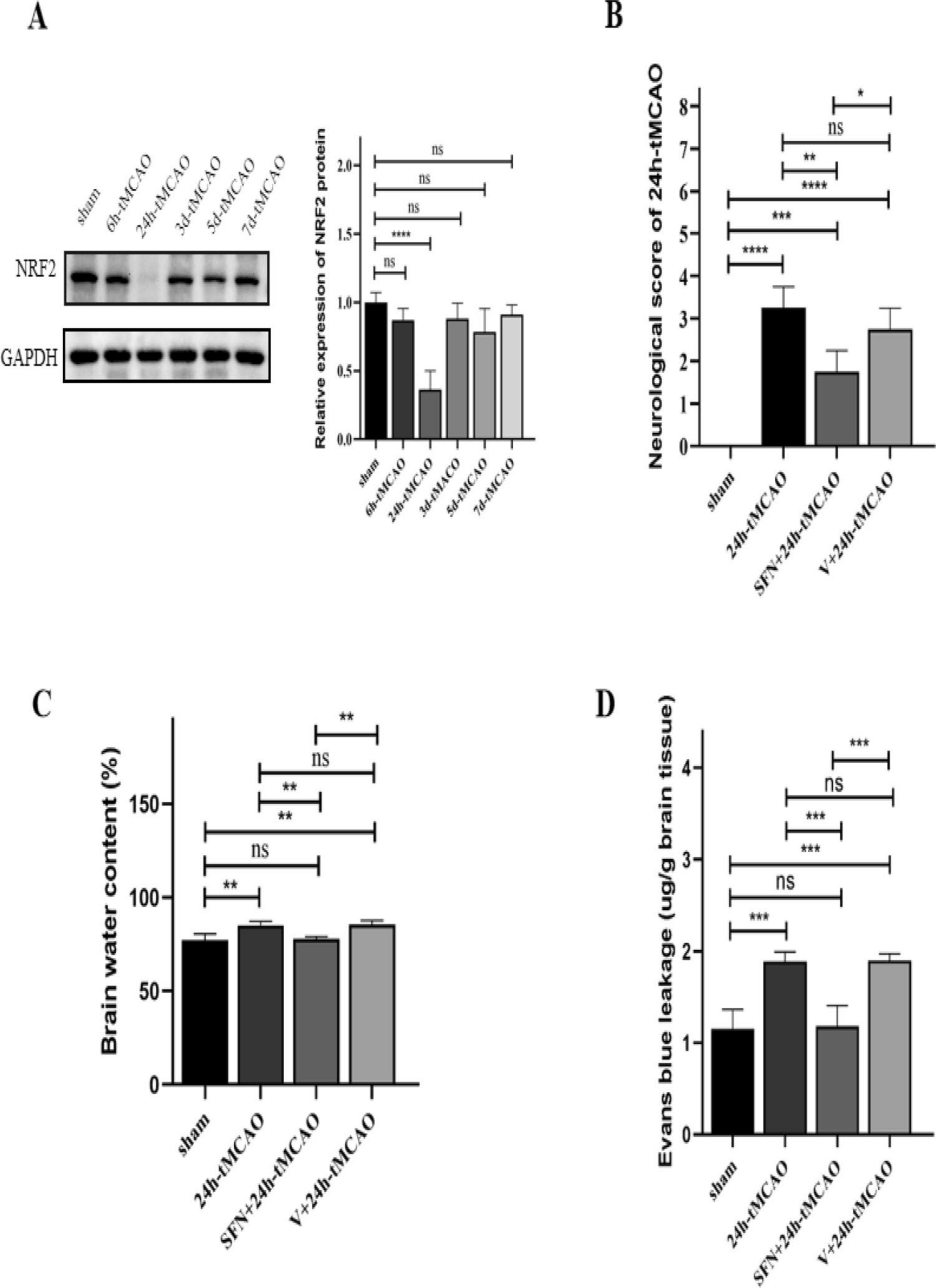
NRF2 expression in diverse time periods within mouse brain tissues post-tMCAO. NRF2 activation ameliorated cerebral ischemic reperfusion injury. (**A**) NRF2 protein expression detected by western blot (n = 4). (**B**) Neurological deficit scores in mice evaluated at 24 h post-tMCAO (n = 4). (**C**) Brain water content measured by the wet/dry approach for assessing brain edema (n = 4). (**D**) Evans blue dye leakage quantified using a spectrophotometer (n = 4). The results are indicated to be means ± SEM. *p < 0.05; **p < 0.01; ***p < 0.001; ****p < 0.0001 and N.S. nonsignificant versus the sham group.

NRF2 was downregulated following tMCAO. To analyze whether NRF2 was associated with cerebral I/R injury, we analyzed NRF2 expression within right mouse brain at diverse time points post-tMCAO. According to WB, NRF2 protein expression decreased over time. NRF2 began to decline in the 6th hour and reached its lowest level (*p* < 0.0001) at 24 h (Fig. 1A) in comparison with the sham group.

### NRF2 activation attenuates cerebral I/R injury in mice

**Figure 2:**
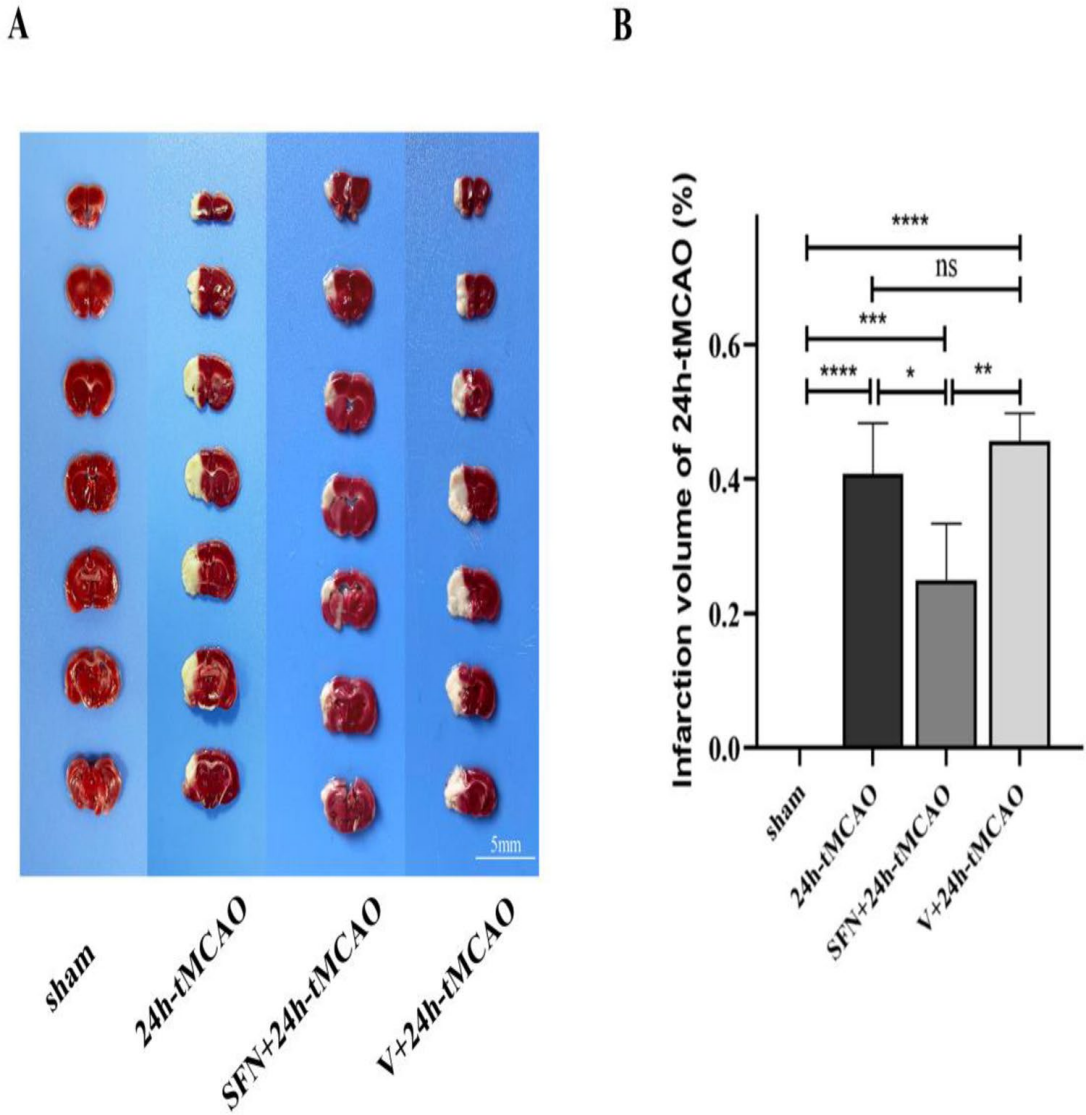
NRF2 activator reduced infarction volume in mice brain tissue post-tMCAO. (**A**, **B**) Typical images showing TTC-stained brain sections and quantification of infarct volume in different groups (n = 4). Results are represented to be means ± SEM. *p < 0.05; **p < 0.01; ***p < 0.001; ****p < 0.0001 and N.S. nonsignificant versus the sham group. Scale bar = 5 mm.

To investigate if SFN conferred the neuroprotection against I/R injury, this study measured neurological deficit score, infarct volume, and brain water content at 24 h post-ischemia. As shown in Fig. 1B, SFN apparently decreased the neurological functions of the brain in relative to 24 h-tMCAO group (*p* < 0.01). To assess the cerebral edema because of cerebral I/R injury, the current study measured brain water content in mice. At 24 h post-tMCAO, brain water content notably increased (*p* < 0.01) in relative to that of sham group (Fig. 1C), and that noticeably decreased in mice of 24 h-tMCAO group given SFN (*p* < 0.01). After SFN injection, lower cellular water content was observed than V + 24 h-tMCAO group (*p* < 0.01). Evans blue staining was performed to analyze BBB infiltration. Relative to the sham group, Evans blue leakage was apparently increased following tMCAO (*p* < 0.001) and reduced following the SFN treatment (*p* < 0.001) (Fig. 1D). Therefore, the NRF2 activator reduced BBB permeability. Compared with 24 h-tMCAO, the mice showed decreased infarction volume after the SFN treatment (*p* < 0.05) (Fig. 2A,B). The obtained findings suggested that SFN protected the brain from I/R damage.

### SFN up-regulated Occludin and ZO1 levels in mice following tMCAO

**Figure 3:**
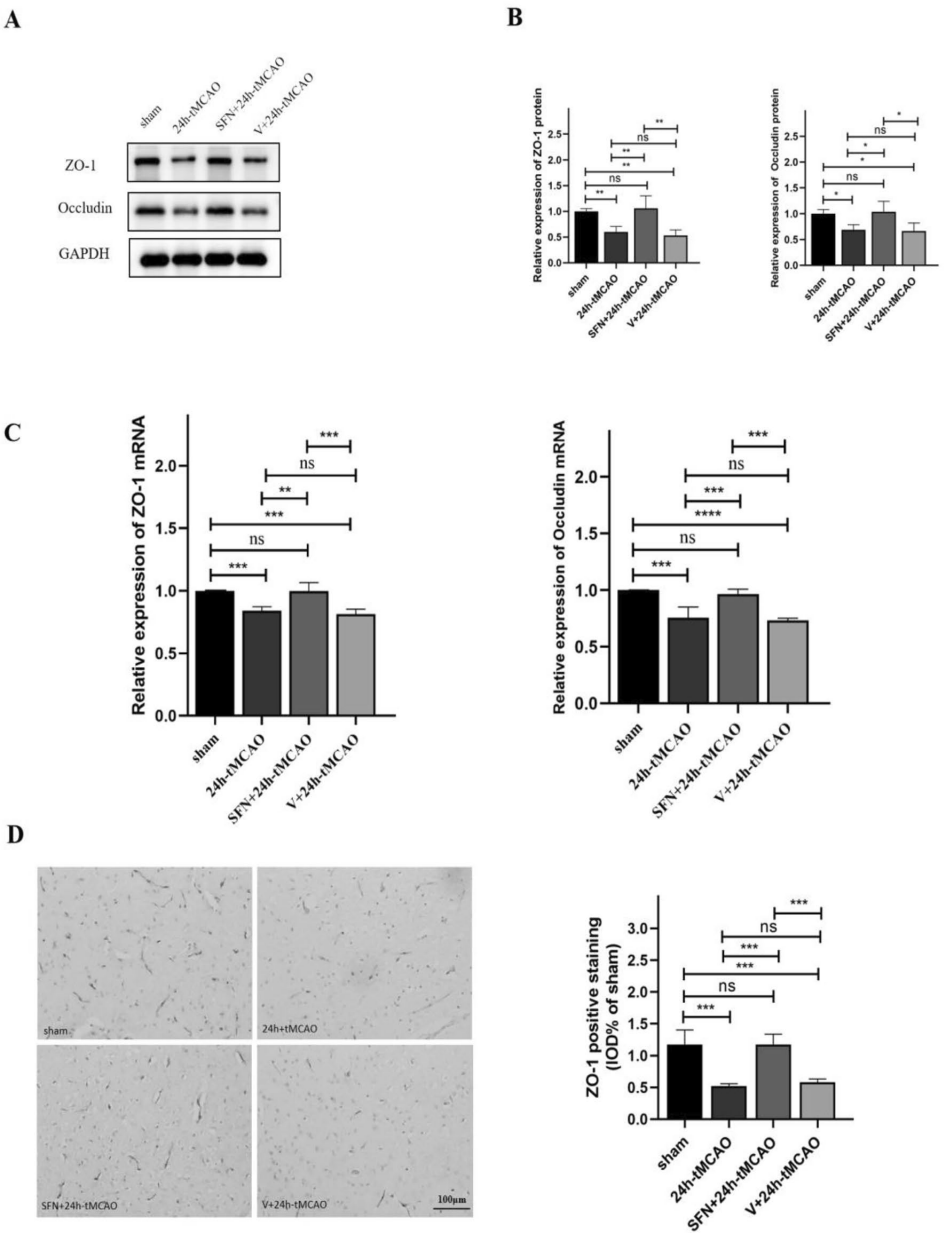
NRF2 activation elevated ZO-1 and occludin levels within mouse brain tissue post-tMCAO. ZO-1 and occludin protein (**A**,**B**) and mRNA (**C**) expression of sham, 24 h-tMCAO, SFN + 24 h tMCAO, and V + 24 h- tMCAO groups (n = 4). (**D**) Immunohistochemical study of ZO-1 in the sham, 24 h-tMCAO, SFN + 24 h tMCAO, and V + 24 h tMCAO groups (n = 4). The results are suggested to be means ± SEM. *p < 0.05; **p < 0.01; ***p < 0.001; ****p < 0.0001 and N.S. nonsignificant versus the sham group. Scale bar = 100 µm.

**Figure 4:**
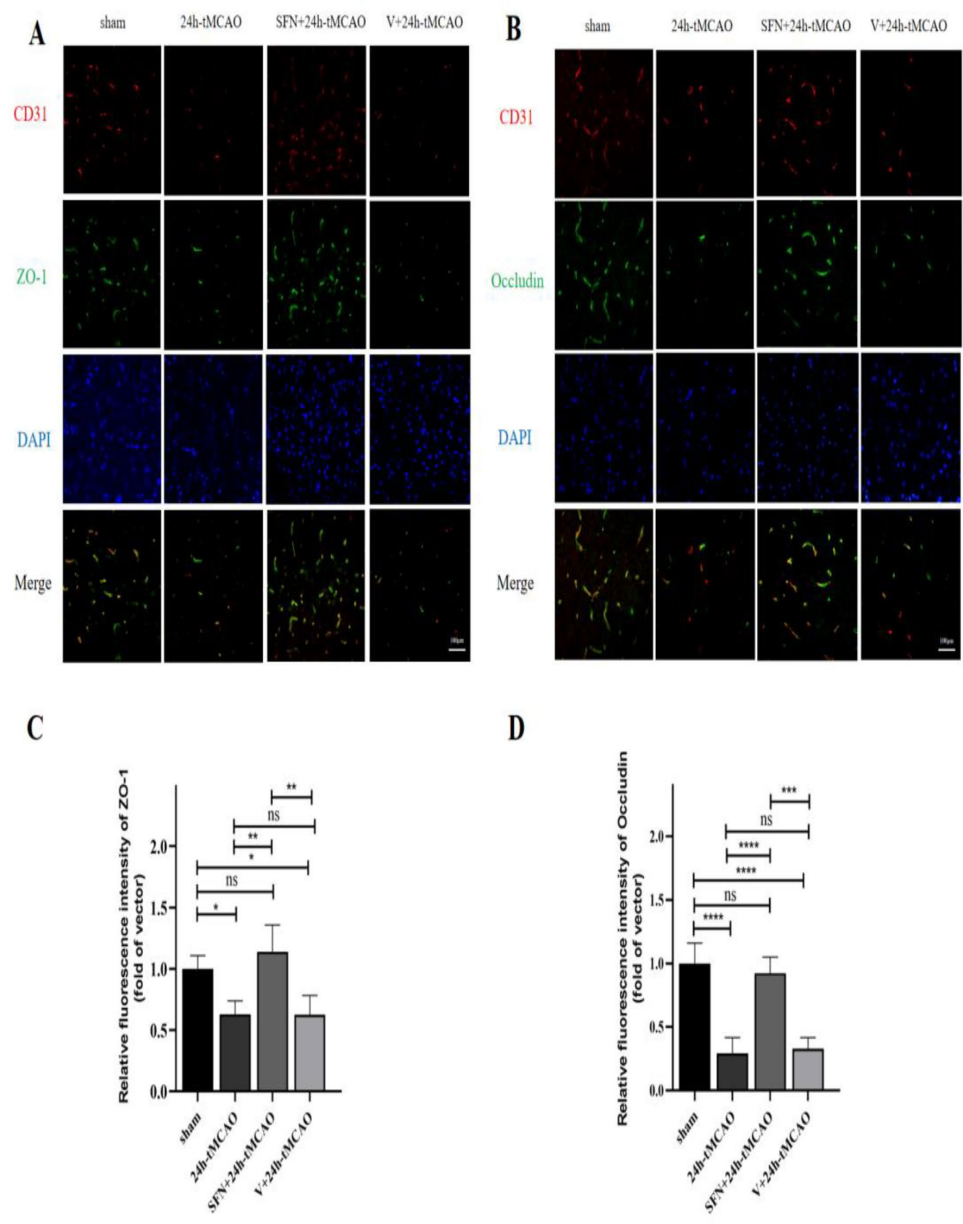
SFN upregulates ZO-1 and occludin levels after tMCAO within mice. (**A**,**B**) Typical images showing double immunofluorescence staining with DAPI (blue)/ZO-1 (green) (**A**) and occludin (green) (**B**)/CD31 (red) colocalization (n = 4). (**C**,**D**) Immunofluorescence analysis of ZO1 and occludin (n = 4). The results indicate means ± SEM. *p < 0.05; **p < 0.01; ***p < 0.001; ****p < 0.0001 and N.S. nonsignificant versus the sham group. Scale bar = 100 µm.

Endothelial cells in CNS-nourishing capillaries are closely connected with each other via tight junctions, which are crucial in keeping the BBB intact^30^. We studied TJ-associated genes and proteins using qRT-PCR and WB. Based on the results, the ZO-1 and occludin protein expression notably reduced at 24-h post-ischemia compared with sham group (*p* < 0.01, *p* < 0.05, respectively). SFN treatment ameliorated MCAO-mediated alterations of the ZO-1 and occludin expression (*p* < 0.01 and* p* < 0.05, separately) (Fig. 3A,B). qRT-PCR results indicated reduced ZO-1 and occludin mRNA expression of MCAO group on the first day (*p* < 0.001), indicating impaired BBB integrity post-IS. SFN upregulated ZO-1 and occludin transcription expression (*p* < 0.01, *p* < 0.001) (Fig. 3C). IOD analysis on ZO-1-positive staining revealed that SFN administration increased ZO-1 positive staining relative to V + 24 h-tMCAO and 24 h-tMCAO treatments (*p* < 0.001) (Fig. 3D). As the fluorescence microscopy analysis indicate, endothelial marker CD31 was co-localized with the TJ proteins, which were used to label the microvessels in mouse brain slices. Consequently, ZO-1 and occludin were coexpressed with CD31, which significantly decreased after 24 h-tMCAO within peri-infarct area (*p* < 0.05, *p* < 0.0001). Nonetheless, the SFN + 24 h-tMCAO group showed significantly increased colocalization relative to the 24 h-tMCAO group (*p* < 0.01,* p* < 0.0001, separately) (Fig. 4C,D), conforming to WB and qRT-PCR assay results. Based on the aforementioned results, SFN preserved BBB integrity following tMCAO.

### SFN decreased MMP9 and AQP4 Levels following tMCAO

**Figure 5:**
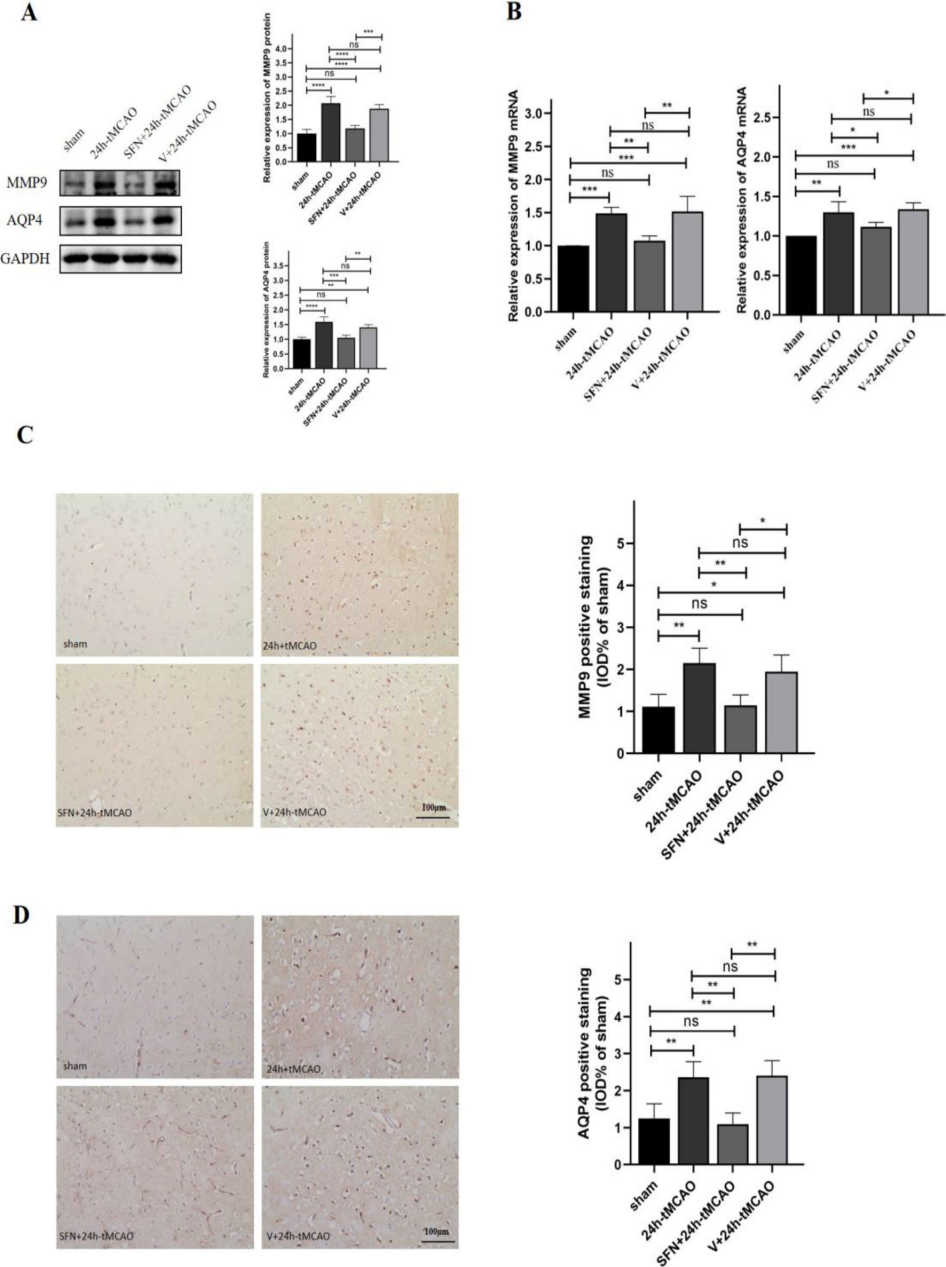
NRF2 activation lowers MMP9 and AQP4 levels within mouse brain tissues post-tMCAO. MMP9 and AQP4 protein (**A**) and mRNA (**B**) expression of sham, 24 h-tMCAO, SFN + 24 h tMCAO, and V + 24 h tMCAO groups (n = 4). Immunohistochemical study of MMP9 (**C**) and AQP4 (**D**) of sham, 24 h-tMCAO, SFN + 24 h tMCAO, and V + 24 h tMCAO groups (n = 4). The results are indicated to be means ± SEM. *p < 0.05; **p < 0.01; ***p < 0.001; ****p < 0.0001 and N.S. nonsignificant versus the sham group. Scale bar = 100 µm.

**Figure 6:**
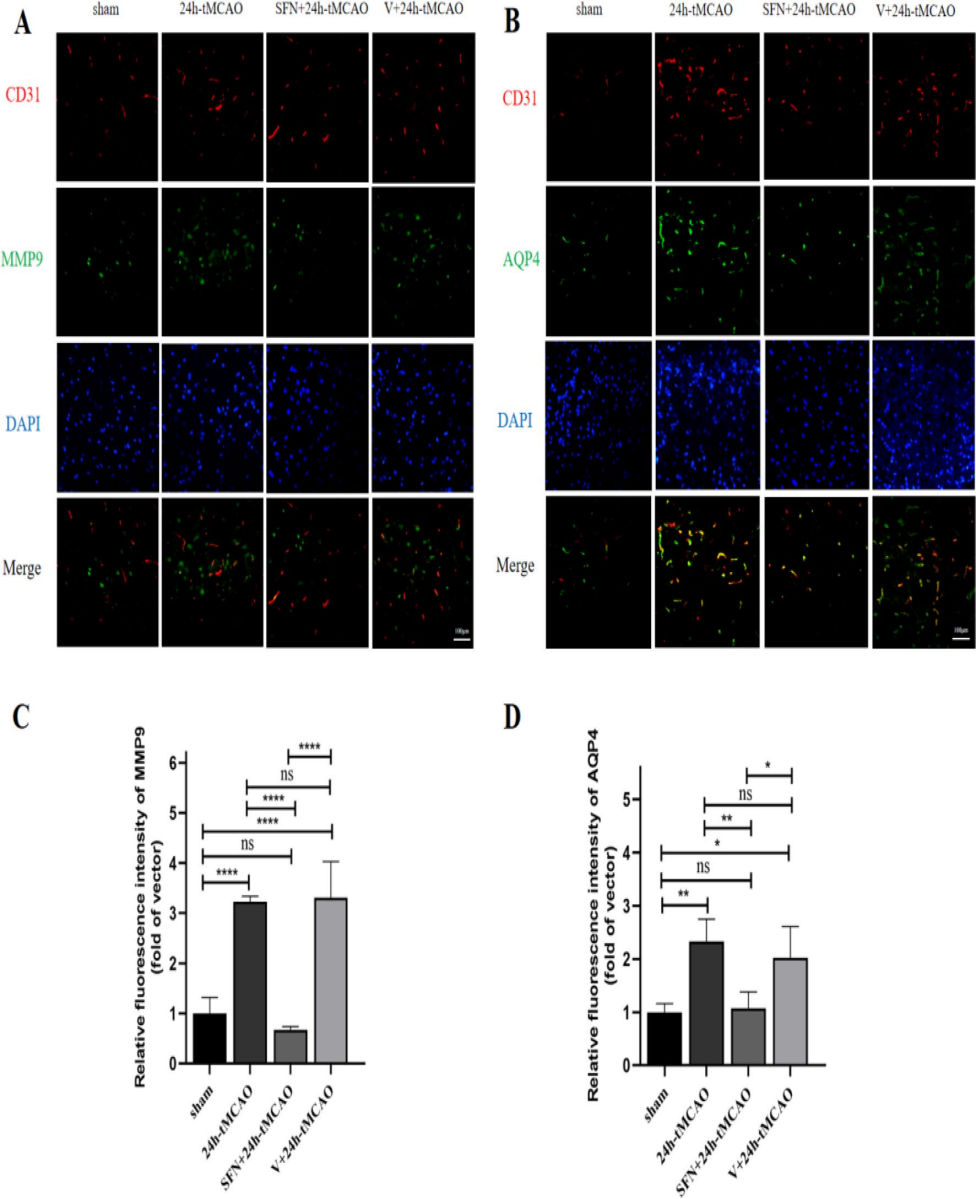
SFN decreased MMP9 and AQP4 levels in mice post-tMCAO. (**A**,**B**) Typical images showing double immunofluorescence staining (n = 4) for DAPI (blue)/MMP9 (green) (A) and AQP4 (green) (B)/CD31 (red) colocalization. (**C**,**D**) Immunofluorescence analysis of MMP9 and AQP4 (n = 4). The results are suggested to be means ± SEM. *p < 0.05; **p < 0.01; ***p < 0.001; ****p < 0.0001 and N.S. nonsignificant versus the sham group. Scale bar = 100 µm.

The dynamic changes in the BBB components, including MMP9 and AQP4, were compared in mice after tMCAO. To assess whether NRF2 activation affects MMP9 and AQP4 in cerebral I/R, WB, qRT-PCR, immunohistochemical staining, and double-immunofluorescence staining were performed. Relative to sham group, WB results indicated that MMP9 and AQP4 of 24 h-tMCAO group were upregulated (*p* < 0.0001). In relative to 24 h-tMCAO and V + 24 h-tMCAO groups, SFN administration decreased MMP9 levels (*p* < 0.0001, *p* < 0.001, separately) (Fig. 5A). Additionally, MMP9 mRNA expression was determined, which suggested that 24 h-tMCAO group showed upregulation relative to sham group (*p* < 0.001), but NRF2 activation decreased MMP9 transcription level (*p* < 0.01) (Fig. 5B). Conforming to the above results, immunohistochemical staining exhibited the same trend for MMP9 in WB **(**Fig. 5C**)**. Meanwhile, after treatment with SFN, the AQP4 protein expression decreased relative to 24 h-tMCAO and V + 24 h-tMCAO groups (*p* < 0.001, *p* < 0.01, separately) (Fig. 5A). In addition, the 24 h-tMCAO group showed elevated AQP4 mRNA transcript expression in comparison with sham group (*p* < 0.01); however, NRF2 activation decreased AQP4 transcript expression (*p* < 0.05) (Fig. 5B). Meanwhile, the immunohistochemical analysis of AQP4 showed the same trend (Fig. 5D). Increased MMP9 expression in I/R was related to the compromised BBB integrity^31^. Relative to sham group, the immunofluorescence of MMP9 surrounding cerebral ischemic area in 24 h-MCAO group was remarkably elevated, while that in SFN + 24 h-tMCAO group apparently declined (*p* < 0.0001) (Fig. 6C**)**. AQP4, the water channel protein that can be detected in astrocyte end feet within the brain, can be applied in observing BBB dysregulation^32^. According to Fig. 6D, AQP4 shows colocalization with CD31 within mouse brain tissues, and double-immunofluorescence was observed along the blood vessels. Relative to the sham group, AQP4 distribution around the blood vessels increased after 24 h-tMCAO (*p* < 0.01). After SFN injection, the immunofluorescence of AQP4 within the peripheral region of the blood vessels decreased (*p* < 0.01), suggesting that NRF2 activation was related to AQP4 deregulation (Fig. 6D). Combinedly, these results showed that SFN reduces BBB permeability among stroke mice.

### NRF2 regulates BBB Damage by inhabiting ferroptosis and inflammation

**Figure 7:**
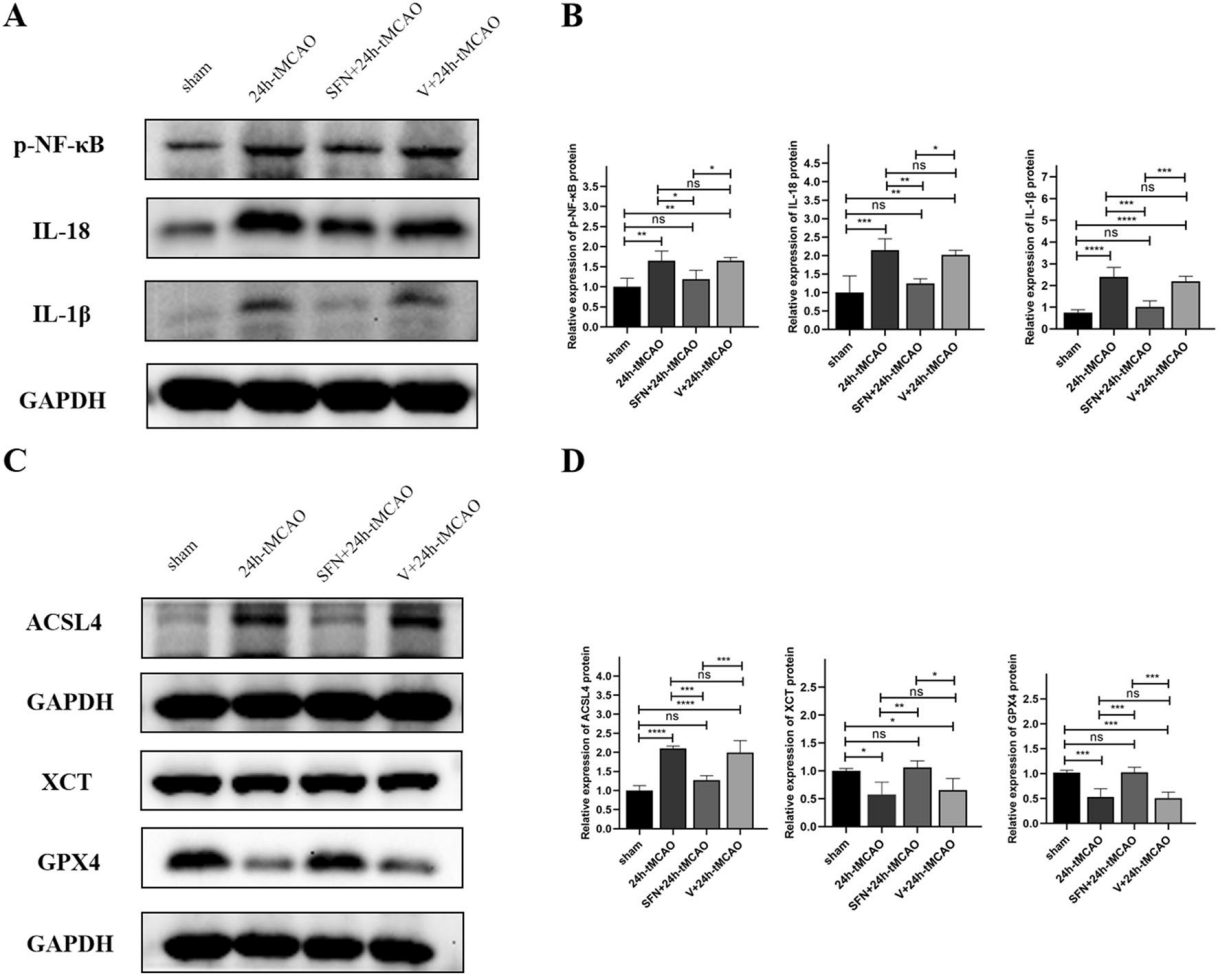
SFN alleviated BBB disruption and brain damage by inhibiting inflammation and ferroptosis. (**A**,**B**) The p-NF-κB, IL-18, and IL-1β protein expression was measured with the application of WB (n = 4). (**C**,**D**) ACSL4, XCT, and GPX4 protein expression was measured with the application of WB (n = 4). Results are indicated to be means ± SEM. *p < 0.05; **p < 0.01; ***p < 0.001; ****p < 0.0001 and N.S. nonsignificant versus the sham group.

**Figure 8:**
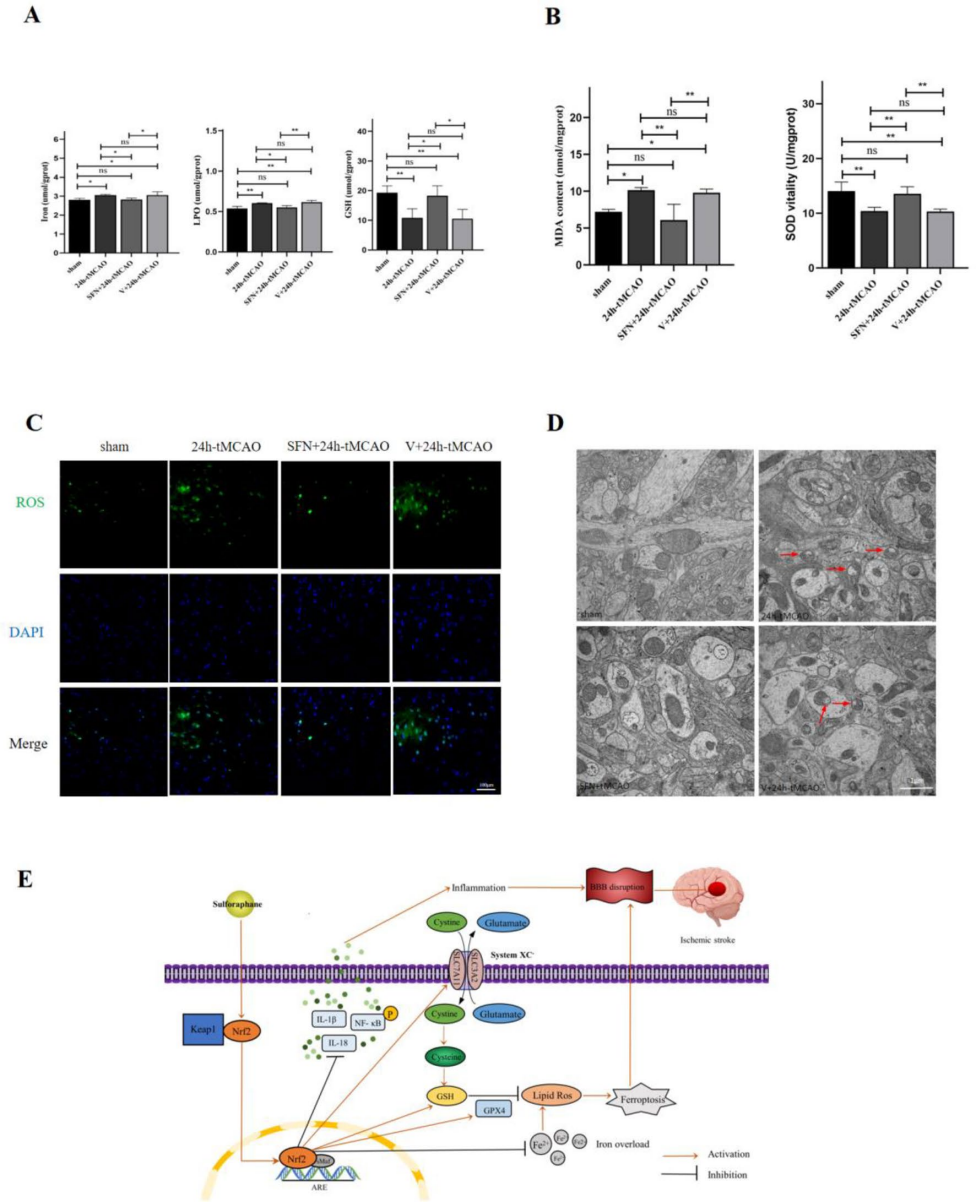
NRF2 activation suppressed ferroptosis in cerebral I/R mice. (**A**,**B**) Iron, LPO, GSH, MDA, and SOD levels were determined using relevant commercially available kits (n = 4). (**C**) Typical images showing ROS using the fluorescent probe DCFH-DA. Scale bar = 100 µm. (**D**) Typical TEM images in the peri-infarct region 24 h post-cerebral ischemic reperfusion. Red arrows suggest rupture of the neuronal outer mitochondrial membrane rapture and mitochondrial cristae reduction or disappearance. Scale bar = 1 µm. Results are suggested to be means ± SEM. *p < 0.05; **p < 0.01; ***p < 0.001; ****p < 0.0001 and N.S. nonsignificant versus the sham group. (**E**) Schematic of the study. Activation of NRF2 decreases cerebral I/R BBB damage by inhibiting ferroptosis and inflammation.

To determine how SFN affected inflammatory responses in I/R, the p-NF-κB, IL-18, and IL-1β levels were measured using WB. The WB results demonstrated that p-NF-κB, IL-18, and IL-1β expression was drastically elevated within brain tissue after 24 h-tMCAO relative to sham group (*p* = 0.003, *p* = 0.0005, *p* < 0.0001, respectively); however, these effects could be blocked by the NRF2 activator (*p* = 0.0304, *p* = 0.0039, *p* = 0.0001, separately) (Fig. 7A,B). For determining the effect of SFN on ferroptosis regulating BBB in the MCAO mice, several critical factors, including GPX4, ACSL4, and XCT, which are the key proteins related to regulating ferroptosis. Therefore, the current study explored the relevant protein expression following I/R. In relative to the sham group, the expressions of GPX4 and XCT were prominently reduced following 24 h of reperfusion (*p* < 0.001, *p* < 0.05, respectively), and ACSL4 expression was simultaneously increased (*p* < 0.0001). When NRF2 was activated by SFN, the decrease of GPX4 and XCT elevated (*p* < 0.001, *p* < 0.01, separately). In comparison, ACSL4 protein expression reduced (*p* < 0.001) (Fig. 7C,D). Ferroptosis-related factors such as iron, GSH, LPO, MDA, and SOD were detected using the kit. Relative to 24 h-tMCAO group, SFN + 24 h-tMCAO administration notably attenuated the increase in iron, LPO, and MDA levels resulting from IS (*p* < 0.05, *p* < 0.05, *p* < 0.01, separately) (Fig. 8A,B). Relative to 24 h-tMCAO group, the IS-induced reduced GSH and SOD levels were significantly alleviated in the SFN + 24 h-tMCAO group (*p* < 0.05, *p* < 0.01, separately) (Fig. 8A,B). SFN treatment improved ROS accumulation and ferroptotic changes in mitochondrial morphological features relative to the 24 h-tMCAO group (Fig. 8C,D). These findings suggest that BBB was alleviated when SFN was administered due tNRF2 activation inhibiting ferroptosis and the inflammatory response.

## Discussion

IS leads to BBB integrity deterioration and enhances permeability, which later aggravates ischemic brain tissue injury. Endothelial BBB represents the primary interface between the CNS and peripheral blood‑derived cells, significantly affecting normal neuronal activity and CNS homeostasis^33,34^. BBB destruction following cerebral ischemia induces inflammatory cell and factor infiltration, thus causing brain edema^35^. Maintaining BBB integrity is important in maintaining brain function while suppressing unfavorable ischemic progression.

Currently, NRF2 activation is identified as the potential target for cerebrovascular events like ischemic stroke. To explore the effect of NRF2 on cerebral I/R injury, NRF2 expression was identified at diverse time points post-tMCAO. WB revealed that NRF2 protein was decreased in the 24 h-tMCAO (*p* < 0.0001 vs. the sham group). It was also suggested that NRF2 protein expression was lower in the experimental group compared with sham group at 3d-tMCAO, 5d-tMCAO, 7d-tMCAO, but the difference was not significant (*p* = 0.4583, *p* = 0.0603, *p* = 0.7059, respectively). Therefore, brain tissue was extracted at 24 h post-tMCAO. NRF2 exerts a crucial effect on managing excess oxidative stress post-IS. It is related to pathways such as PI3K/AKT, Keap1, NF-κB, and HO-1 and suppresses oxidative stress, resists inflammation, maintains mitochondrial homeostasis, and protects BBB to alleviate cerebral I/R^36–40^. ASIV protects BBB integrity via a process that involves the NRF2 pathway^41^. NOM prevents cerebral I/R-mediated BBB deregulation and neurological deficits via the NRF2 pathway^42^. Therefore, NRF2 is potentially related to regulating BBB injury after IS.

TJs build the physical and metabolic barrier that restricts molecule movement between the brain and blood, thus maintaining cerebral homeostasis^43^. Promoting TJ protein production to increase intercellular contacts is the response of the endothelial cells toward the elevated paracellular permeability and cytoarchitectural changes^44^. ZO-1 and Occludin levels are decreased after experimentally inducing cerebral embolism within the separated rat brain capillaries^45^. Dimethyl fumarate (DMF), an NRF2 activator, prevents TJ destruction and decreases matrix metalloproteinase (MMP) activity within brain tissue. Nonetheless, NRF2 silencing deteriorates TJ ZO-1 delocalization in the case of stroke and reduces DMF protection, suggesting that NRF2 exerts a vital role in protecting BBB integrity^46^. In this study, NRF2 critically affected brain edema and BBB destruction post-tMCAO. Neurological deficits were alleviated, and BBB integrity was improved after the NRF2 treatment. Further analyzing NRF2 as the novel target for developing the anti-cerebral infarction therapeutic target and discovering novel drugs will be a significant future research direction.

In addition, MMP9, a gelatinase belonging to the MMP family, regulates BBB maintenance and structural components, like junctional proteins, extracellular matrix proteins, and basement membrane^47^. In the transient focal cerebral ischemia models, MMP9 mRNA knockdown in rats or MMP9 genetic deletion in mice remarkably decreased edema and BBB disruption, which is related to the lower TJ protein degradation (like ZO-1)^48,49^. MMP9 activation and upregulation lead to deregulated BBB integrity through TJ protein degradation and inflammation modulation^50,51^. The MMP up-regulation time course, particularly MMP9, is closely related to the maximum BBB disruption^52^^.^

AQP4, a water channel protein, shows polarized expression in the astroglial endfeet around brain microvessels, which affects brain water balance^53^. Suppressing AQP4 via TGN-020 enhances neurological recovery by suppressing brain edema in the early stage and mitigating AQP4 depolarization and peri-infarct astrogliosis in the subacute stage post-stroke^54^. AQP4 expression is significantly increased within the brain post-cerebral infarction^55,56^. In MCAO animals, AQP4 silencing alone decreased the infarction volume while improving neurological activity and neuronal survival^57,58^. Herein, AQP4 mRNA and protein expression increased post-tMCAO. The immunofluorescence analysis revealed that AQP4 expression surrounding blood vessels was up-regulated following cerebral ischemia. After activating NRF2, AQP4 expression was inhibited, illustrating the involvement of NRF2 in AQP4 downregulation.

Inflammatory factors like TNF-α activate NF-κB and MMPs to destroy BBB^59,60^. Proinflammatory cytokines TNF-α, NO, IL-1β, and prostaglandins are related to BBB integrity disruption in IS^61,62^. TNF-α and IL-1β promote AQP4 expression within the IS model, generating the positive feedback loop for amplifying brain injury in IS^63–65^. DMF, the NRF2 activator directly contributing to sustaining endothelial TJs, suppresses inflammatory factor levels^46^. In addition, tert-butyl hydroquinol (t BHQ), an NRF2 activator, remarkably decreases caspase-1, IL-18, and IL-1β levels post-tMCAO^66^. Ferroptosis, the novel regulatory cell death type, results from excess lipid peroxide and iron-dependent ROS accumulation. Lipid peroxidation, iron accumulation, and increased ROS are related to barrier dysfunction^67^. Our study showed that the SFN treatment alleviated BBB disruption and brain damage by inhibiting inflammation and ferroptosis. SFN protects against neurological deficits and BBB disruption during IS^16,68^. However, further studies on protecting BBB via NRF2 are necessary. SFN represents the strong natural NRF2 activator with neuroprotection and BBB permeability during acute stroke. Figure 8E shows the schematic. This study has several limitations as well. First, when we constructed the tMCAO model, doppler was not used to comfirm MCAO. What's more, since the small sample and known surgical variability size, we should increase a greater number of specimens to prove our study (S1). Besides, more clinical pathology samples of human patients with ischemic stroke are further needed to verify the role of NRF2 in the future.

## Conclusion

To conclude, the present study showed that the upregulated NRF2 protects against BBB integrity destruction during cerebral I/R injury via regulating of inflammation and ferroptosis. Moreover, the findings of the current study highlight the novel possible therapeutic targets and mechanisms underlying BBB destruction after cerebral I/R injury. Combinedly, SFN, a specific activator of NRF2, may become an effective therapeutic approach for cerebral ischemia and reperfusion.

### Supplementary Information

Supplementary Figures.

## Data Availability

The original contributions presented in the study are included in the article material, further inquiries can be directed to the corresponding author/s.

## References

[1] Feigin VL, Stark BA (2021). Global, regional, and national burden of stroke and its risk factors, 1990–2019: A systematic analysis for the global burden of disease study 2019. Lancet Neurol..

[2] Saini V, Guada L, Yavagal DR (2021). Global epidemiology of stroke and access to acute ischemic stroke interventions. Neurology.

[3] Donkor ES (2018). Stroke in the 21st century: A snapshot of the burden, epidemiology, and quality of life. Stroke Res. Treat..

[4] Daneman R, Engelhardt B (2017). Brain barriers in health and disease. Neurobiol. Dis..

[5] Ronaldson PT, Davis TP (2020). Regulation of blood-brain barrier integrity by microglia in health and disease: A therapeutic opportunity. J. Cereb. Blood Flow Metab..

[6] Fang W, Sha L, Kodithuwakku ND (2015). Attenuated blood–brain barrier dysfunction by XQ-1H following ischemic stroke in hyperlipidemic rats. Mol. Neurobiol..

[7] Nguyen T, Nioi P, Pickett CB (2009). The Nrf2-antioxidant response element signaling pathway and its activation by oxidative stress. J. Biol. Chem..

[8] Vomhof-dekrey EE, Picklo MJ (2012). The Nrf2-antioxidant response element pathway: A target for regulating energy metabolism. J. Nutr. Biochem..

[9] Li W, Khor TO, Xu C (2008). Activation of Nrf2-antioxidant signaling attenuates NFκB-inflammatory response and elicits apoptosis. Biochem. Pharmacol..

[10] Dinkova-kostova AT, Talalay P (2010). NAD(P)H:quinone acceptor oxidoreductase 1 (NQO1), a multifunctional antioxidant enzyme and exceptionally versatile cytoprotector. Arch. Biochem. Biophys..

[11] Khan M, Dhammu TS, Sakakima H (2012). The inhibitory effect of S-nitrosoglutathione on blood–brain barrier disruption and peroxynitrite formation in a rat model of experimental stroke. J. Neurochem..

[12] Zhao X, Li S, Mo Y (2021). DCA protects against oxidation injury attributed to cerebral ischemia-reperfusion by regulating glycolysis through PDK2-PDH-Nrf2 axis. Oxidative Med. Cell. Longev..

[13] Ng AYH, Li Z (2019). Regulator of G protein signaling 12 enhances osteoclastogenesis by suppressing Nrf2-dependent antioxidant proteins to promote the generation of reactive oxygen species. Elife.

[14] Zhuang Y, Wu H, Wang X (2019). Resveratrol attenuates oxidative stress-induced intestinal barrier injury through PI3K/Akt-mediated Nrf2 signaling pathway. Oxidative Med. Cell. Longev..

[15] Sun Y-Y, Zhu H-J, Zhao R-Y (2023). Remote ischemic conditioning attenuates oxidative stress and inflammation via the Nrf2/HO-1 pathway in MCAO mice. Redox Biol..

[16] Alfieri A, Srivastava S, Siow RCM (2013). Sulforaphane preconditioning of the Nrf2/HO-1 defense pathway protects the cerebral vasculature against blood–brain barrier disruption and neurological deficits in stroke. Free Radic. Biol. Med..

[17] Tuo Q-Z, Zhang S-T, Lei P (2022). Mechanisms of neuronal cell death in ischemic stroke and their therapeutic implications. Med. Res. Rev..

[18] Tuo Q-Z, Lei P, Jackman KA (2017). Tau-mediated iron export prevents ferroptotic damage after ischemic stroke. Mol. Psychiatry.

[19] Chen J, Yang L, Geng L (2021). Inhibition of acyl-CoA synthetase long-chain family member 4 facilitates neurological recovery after stroke by regulation ferroptosis. Front. Cell. Neurosci..

[20] Kong Y, Hu L, Lu K (2019). Ferroportin downregulation promotes cell proliferation by modulating the Nrf2-miR-17-5p axis in multiple myeloma. Cell Death Dis..

[21] Yuan Y, Zhai Y, Chen J (2021). Kaempferol ameliorates oxygen-glucose deprivation/reoxygenation-induced neuronal ferroptosis by activating Nrf2/SLC7A11/GPX4 axis. Biomolecules.

[22] Dodson M, Castro-portuguez R, Zhang DD (2019). NRF2 plays a critical role in mitigating lipid peroxidation and ferroptosis. Redox Biol..

[23] Shahcheraghi SH, Salemi F, Small S (2023). Resveratrol regulates inflammation and improves oxidative stress via Nrf2 signaling pathway: Therapeutic and biotechnological prospects. Phytother. Res. PTR.

[24] Xia M, Zhang Y, Wu H (2022). Forsythoside B attenuates neuro-inflammation and neuronal apoptosis by inhibition of NF-κB and p38-MAPK signaling pathways through activating Nrf2 post spinal cord injury. Int. Immunopharmacol..

[25] Li W, Suwanwela NC, Patumraj S (2016). Curcumin by down-regulating NF-kB and elevating Nrf2, reduces brain edema and neurological dysfunction after cerebral I/R. Microvasc. Res..

[26] Zhang Q, Yin J, Xu F (2021). Isoflurane post-conditioning contributes to anti-apoptotic effect after cerebral ischaemia in rats through the ERK5/MEF2D signaling pathway. J. Cell. Mol. Med..

[27] She DT, Wong LJ, Baik S-H (2018). SIRT2 inhibition confers neuroprotection by downregulation of FOXO3a and MAPK signaling pathwaysin ischemic stroke. Mol. Neurobiol..

[28] Zhu W, Davis CM, Allen EM (2023). Sex difference in capillary reperfusion after transient middle cerebral artery occlusion in diabetic mice. Stroke.

[29] Chen S, Peng J, Sherchan P (2020). TREM2 activation attenuates neuroinflammation and neuronal apoptosis via PI3K/Akt pathway after intracerebral hemorrhage in mice. J. Neuroinflamm..

[30] Alonso-Alonso ML, Sampedro-viana A, Fernández-rodicio S (2022). Need for a paradigm shift in the treatment of ischemic stroke: The blood–brain barrier. Int. J. Mol. Sci..

[31] Fujimura M (1999). Early appearance of activated matrix metalloproteinase-9 and blood–brain barrier disruption in mice after focal cerebral ischemia and reperfusion. Brain Res..

[32] Dunn C, Sturdivant N, Venier S (2021). Blood–brain barrier breakdown and astrocyte reactivity evident in the absence of behavioral changes after repeated traumatic brain injury. Neurotrauma Rep..

[33] Abbott NJ, Patabendige AAK, Dolman DEM (2010). Structure and function of the blood–brain barrier. Neurobiol. Dis..

[34] Kadry H, Noorani B, Cucullo L (2020). A blood–brain barrier overview on structure, function, impairment, and biomarkers of integrity. Fluids Barriers CNS.

[35] Jiang X, Andjelkovic AV, Zhu L (2018). Blood–brain barrier dysfunction and recovery after ischemic stroke. Prog. Neurobiol..

[36] Guo L, Shi L (2023). Vitexin improves cerebral ischemia-reperfusion injury by attenuating oxidative injury and ferroptosis via Keap1/Nrf2/HO-1signaling. Neurochem. Res..

[37] Zhang Z, Xu C, Hao J (2020). Beneficial consequences of Lupeol on middle cerebral artery-induced cerebral ischemia in the rat involves Nrf2 and P38 MAPK modulation. Metab. Brain Dis..

[38] Zhang W, Song J-K, Yan R (2018). Diterpene ginkgolides protect against cerebral ischemia/reperfusion damage in rats by activating Nrf2 and CREB through PI3K/Akt signaling. Acta Pharmacol. Sin..

[39] Gao Y, Hu M, Niu X (2022). Dl-3-n-butylphthalide improves neuroinflammation in mice with repeated cerebral ischemia-reperfusion injury through the Nrf2-mediated antioxidant response and TLR4/MyD88/NF-κB signaling pathway. Oxidative Med. Cell. Longev..

[40] Wang J, Zhang W, Lv C (2020). A novel biscoumarin compound ameliorates cerebral ischemia reperfusion-induced mitochondrial oxidative injury via Nrf2/Keap1/ARE signaling. Neuropharmacology.

[41] Li H, Wang P, Huang F (2018). Astragaloside IV protects blood–brain barrier integrity from LPS-induced disruption via activating Nrf2 antioxidant signaling pathway in mice. Toxicol. Appl. Pharmacol..

[42] Shi Y-S, Zhang Y, Liu B (2019). Nomilin protects against cerebral ischemia-reperfusion induced neurological deficits and blood–brain barrier disruption via the Nrf2 pathway. Food Funct..

[43] Huber JD, Egleton RD, Davis TP (2001). Molecular physiology and pathophysiology of tight junctions in the blood–brain barrier. Trends Neurosci..

[44] Wolburg H, Lippoldt A (2002). Tight junctions of the blood–brain barrier: Development, composition and regulation. Vascul. Pharmacol..

[45] Kago T, Takagi N, Date I (2006). Cerebral ischemia enhances tyrosine phosphorylation of occludin in brain capillaries. Biochem. Biophys. Res. Commun..

[46] Kunze R, Urrutia A, Hoffmann A (2015). Dimethyl fumarate attenuates cerebral edema formation by protecting the blood–brain barrier integrity. Exp. Neurol..

[47] Ramos-fernandez M, Bellolio MF, Stead LG (2011). Matrix metalloproteinase-9 as a marker for acute ischemic stroke: A systematic review. J. Stroke Cerebrovasc. Dis..

[48] Asahi M (2001). Effects of matrix metalloproteinase-9 gene knock-out on the proteolysis of blood–brain barrier and white matter components after cerebral ischemia. J. Neurosci..

[49] Hu Q, Chen C, Khatibi NH (2011). Lentivirus-mediated transfer of MMP-9 shRNA provides neuroprotection following focal ischemic brain injury in rats. Brain Res..

[50] Yang C, Hawkins KE, Doré S (2019). Neuroinflammatory mechanisms of blood–brain barrier damage in ischemic stroke. Am. J. Physiol. Cell Physiol..

[51] Candelario-jalil E, Dijkhuizen RM, Magnus T (2022). Neuroinflammation, stroke, blood–brain barrier dysfunction, and imaging modalities. Stroke.

[52] Shigemori Y (2006). Matrix metalloproteinase 9 is associated with blood brain barrier opening and brain edema formation after cortical contusion in rats. Acta Neurochir. Suppl..

[53] Vandebroek A, Yasui M (2020). Regulation of AQP4 in the central nervous system. Int. J. Mol. Sci..

[54] Sun C, Lin L, Yin L (2022). Acutely inhibiting AQP4 with TGN-020 improves functional outcome by attenuating edema and peri-infarct astrogliosis after cerebral ischemia. Front. Immunol..

[55] Zador Z (2009). Role of aquaporin 4 in cerebral edema and stroke. Handb. Exp. Pharmacol..

[56] Manley GT (2000). Aquaporin-4 deletion in mice reduces brain edema after acute water intoxication and ischemic stroke. Nat. Med..

[57] Yang C, Liu Z, Li H (2015). Aquaporin-4 knockdown ameliorates hypoxic-ischemic cerebral edema in newborn piglets. IUBMB Life.

[58] Tourdias T (2011). Differential aquaporin 4 expression during edema build-up and resolution phases of brain inflammation. J. Neuroinflamm..

[59] Hosomi N, Ban CR (2005). Tumor necrosis factor-alpha neutralization reduced cerebral edema through inhibition of matrix metalloproteinase production after transient focal cerebral ischemia. J. Cereb. Blood Flow Metab..

[60] Brown RC, Mark KS, Egleton RD (2003). Protection against hypoxia-induced increase in blood–brain barrier permeability: Role of tight junction proteins and NFκB. J. Cell Sci..

[61] Zoppo GD (2000). Inflammation and stroke: Putative role for cytokines, adhesion molecules and iNOS in brain response to ischemia. Brain Pathol..

[62] Stanimirovic D, Satoh K (2000). Inflammatory mediators of cerebral endothelium: A role in ischemic brain inflammation. Brain Pathol..

[63] Tang G, Yang G-Y (2016). Aquaporin-4: A potential therapeutic target for cerebral edema. Int. J. Mol. Sci..

[64] Lu H, Ai L, Zhang B (2022). TNF-α induces AQP4 overexpression in astrocytes through the NF-κB pathway causing cellular edema and apoptosis. Biosci. Rep..

[65] Ito H, Yamamoto N, Arima H (2006). Interleukin-1beta induces the expression of aquaporin-4 through a nuclear factor-kappaB pathway in rat astrocytes. J. Neurochem..

[66] Hou Y, Wang Y, He Q (2018). Nrf2 inhibits NLRP3 inflammasome activation through regulating Trx1/TXNIP complex in cerebral ischemia reperfusion injury. Behav. Brain Res..

[67] Chen X, Pang X, Yeo AJ (2022). The molecular mechanisms of ferroptosis and its role in blood–brain barrier dysfunction. Front. Cell. Neurosci..

[68] Mao L, Yang T, Li X (2019). Protective effects of sulforaphane in experimental vascular cognitive impairment: Contribution of the Nrf2 pathway. J. Cereb. Blood Flow Metab..

