# Supplementary material for: NRF2 activation ameliorates blood–brain barrier injury after cerebral ischemic stroke by regulating ferroptosis and inflammation
Source: Sci Rep. 2024 Mar 4;14:5300. doi: 10.1038/s41598-024-53836-0 (PMC10912757; doi:10.1038/s41598-024-53836-0)

NRF2 activation ameliorates blood–brain barrier injury after cerebral ischemic stroke by regulating ferroptosis and inflammation

Wei Fan[1], Hongping Chen[1], Meng Li[1], Xuehui Fan[1], Fangchao Jiang[1], Chen Xu[1], Yingju Wang[1], Wan Wei[1], Jihe Song[1], Di Zhong[1*] and Guozhong Li [1,2*]

[1]Department of Neurology, the First Affiliated Hospital of Harbin Medical University, 23 You Zheng Street, Harbin 150001, Heilongjiang Province, PR China

[2]Department of Neurology, Heilongjiang Provincial Hospital, 82 Zhong Shan Street, Harbin 150001, Heilongjiang Province, PR China

*Corresponding author: Guozhong Li (lgzhyd1962@163.com);

*Corresponding author: Di Zhong (sjnkzhongdi@163.com).

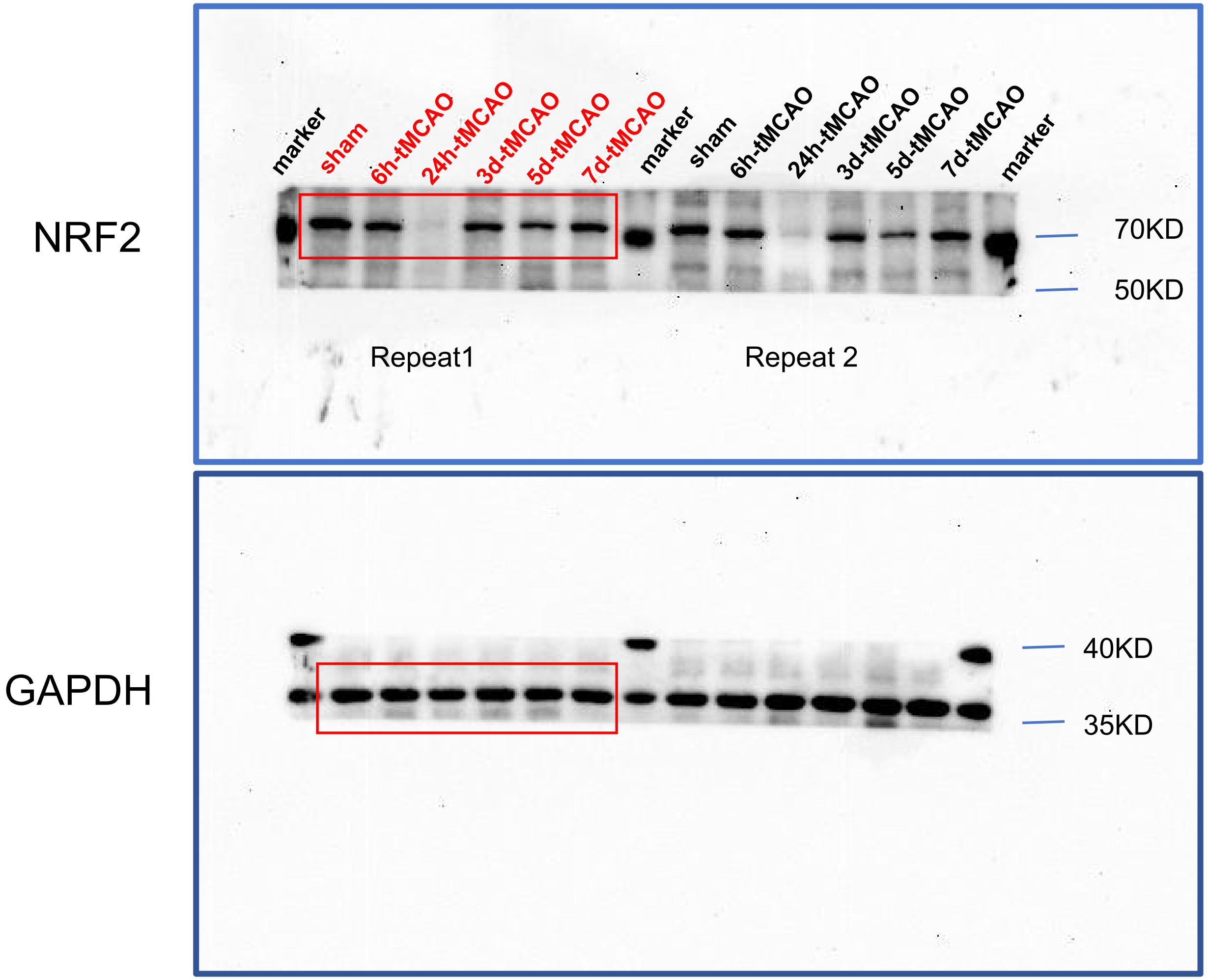

The order of gel stripes in the red box is sham, 6h-tMCAO, 24h-tMCAO, 3d-tMCAO, 5d-tMCAO, 7d-tMCAO.

Supplementary Figure S1. Original Western blots of Figure 1A (red box circled). We completed the Western-blot experiment shown in this picture in the same gel. However, in order to incubate different primary antibodies, western blots were cropped prior to incubation with primary antibody hybridization.. We repeated the experiment three times on a single gelatin plate.

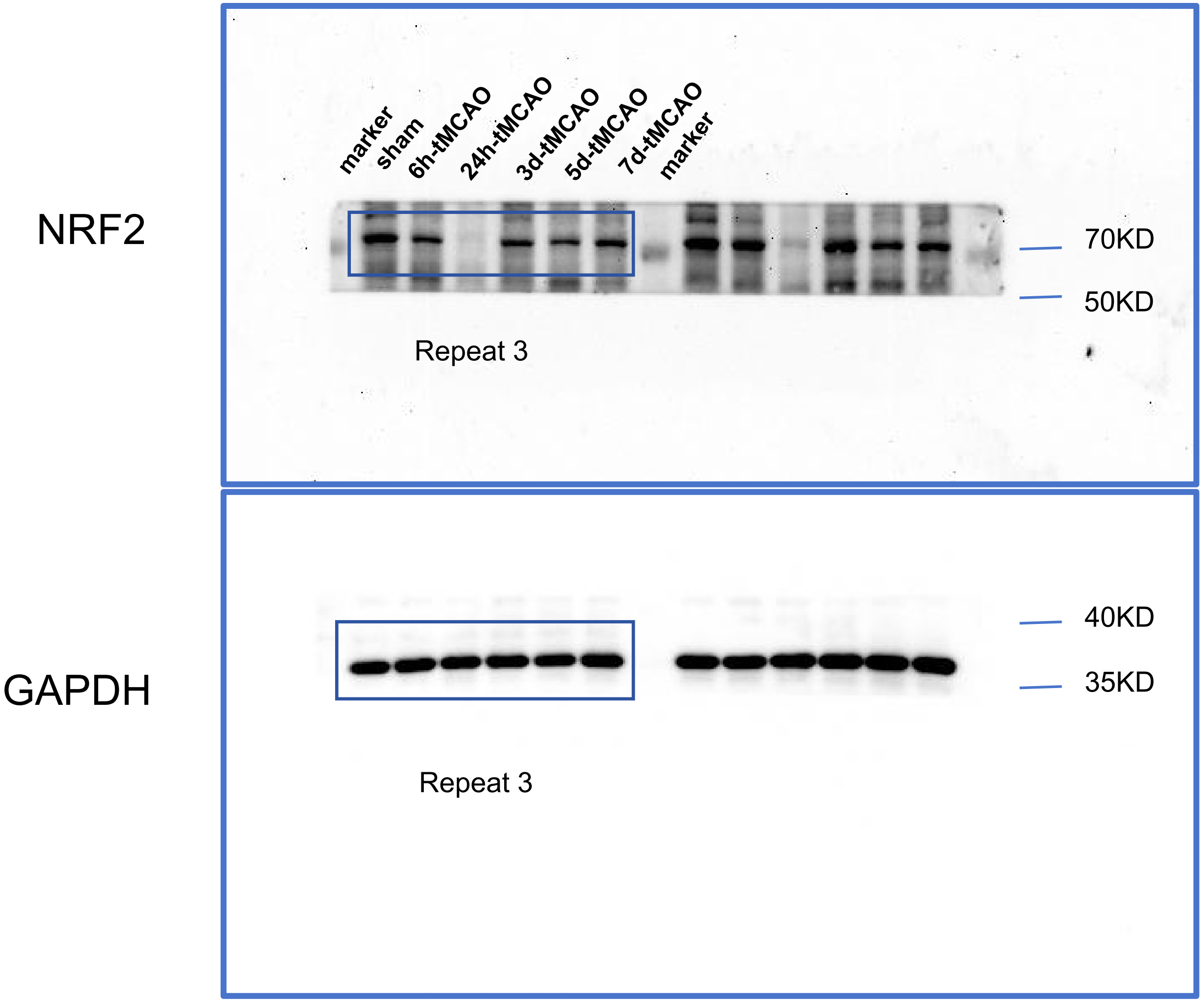

The order of gel stripes in the blue box is sham, 6h-tMCAO, 24h-tMCAO, 3d-tMCAO, 5d-tMCAO, 7-tMCAO.

Supplementary Figure S2. Original Western blots of Figure 1A. We completed the Western-blot experiment shown in this picture in the same gel. However, in order to incubate different primary antibodies, western blots were cropped prior to incubation with primary antibody hybridization.. We repeated the experiment three times on a single gelatin plate.

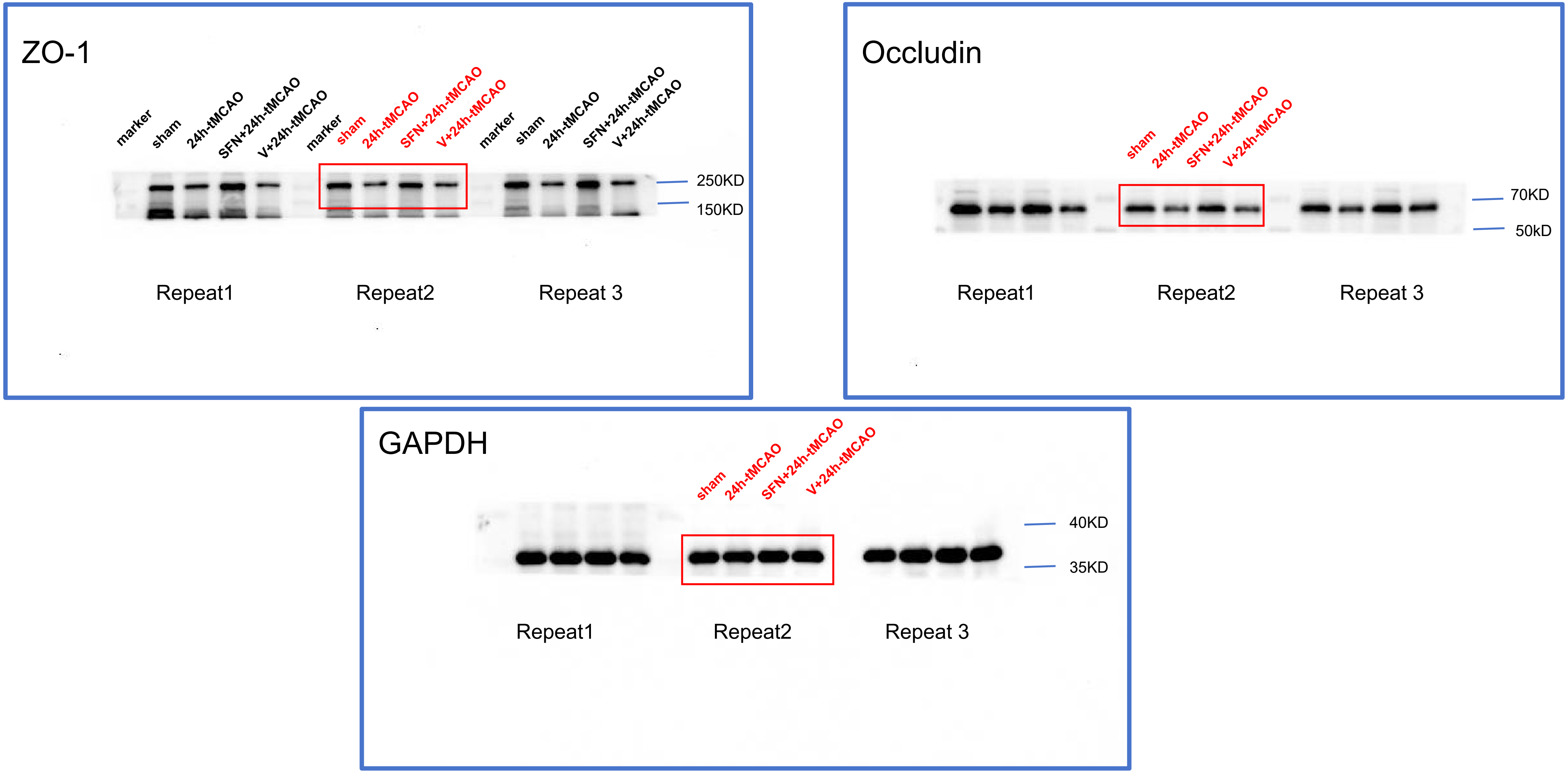

Supplementary Figure S3. Original Western blots of Figure 3A (red box circled). We completed the Western-blot experiment shown in this picture in the same gel. However, in order to incubate different primary antibodies, western blots were cropped prior to incubation with primary antibody hybridization.. We repeated the experiment three times on a single gelatin plate.

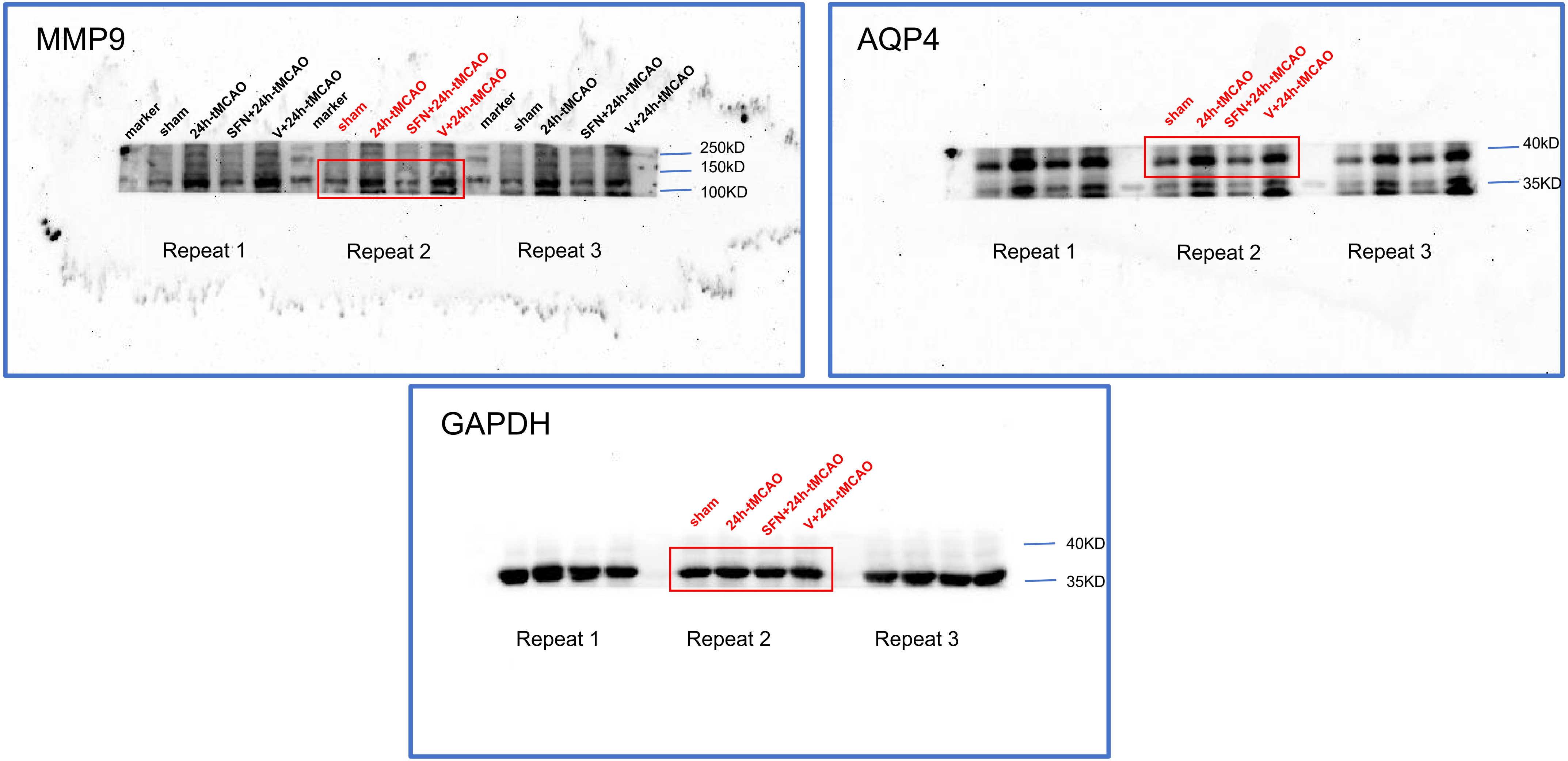

Supplementary Figure S4. Original Western blots of Figure 5A (red box circled). We completed the Western-blot experiment shown in this picture in the same gel. However, in order to incubate different primary antibodies, western blots were cropped prior to incubation with primary antibody hybridization.. We repeated the experiment three times on a single gelatin plate.

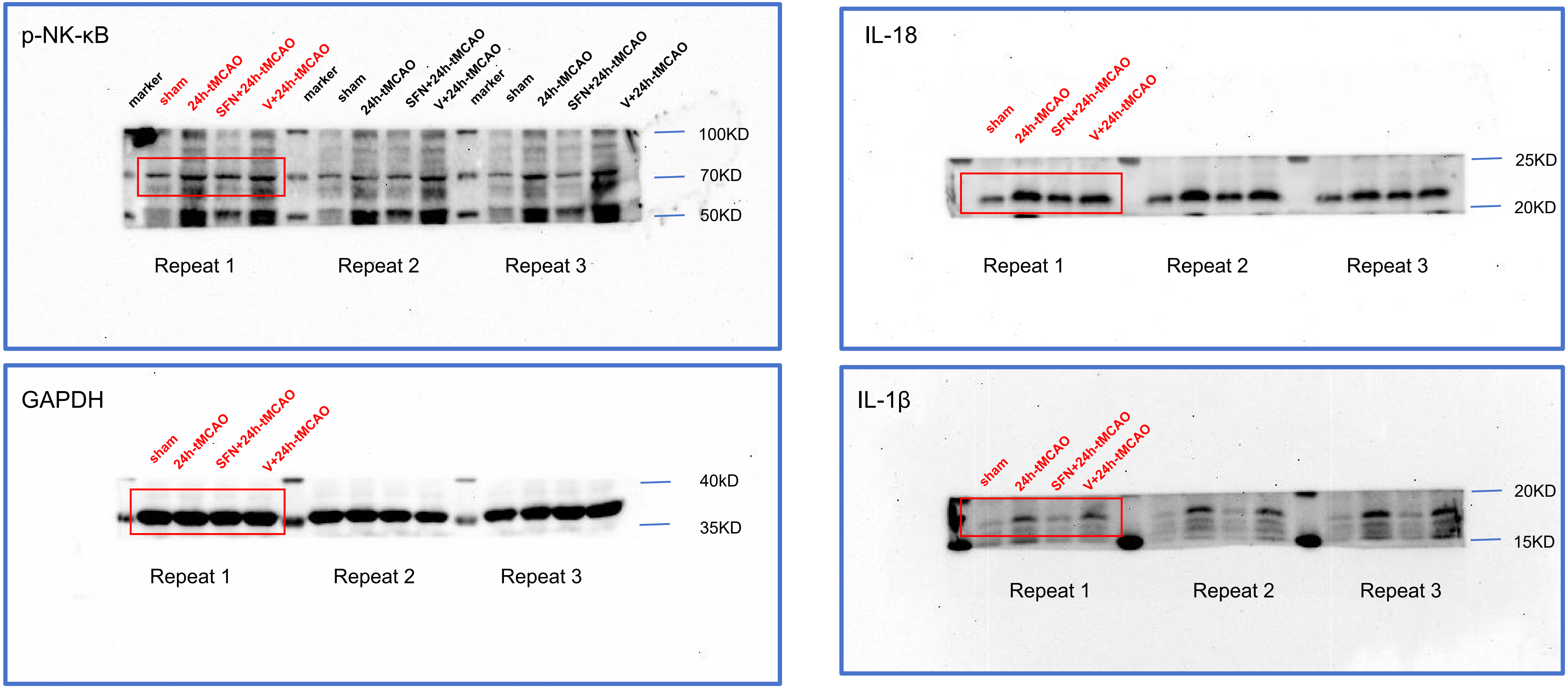

Supplementary Figure S5. Original Western blots of Figure 7A (red box circled). We completed the Western-blot experiment shown in this picture in the same gel. However, in order to incubate different primary antibodies, western blots were cropped prior to incubation with primary antibody hybridization.. We repeated the experiment three times on a single gelatin plate.

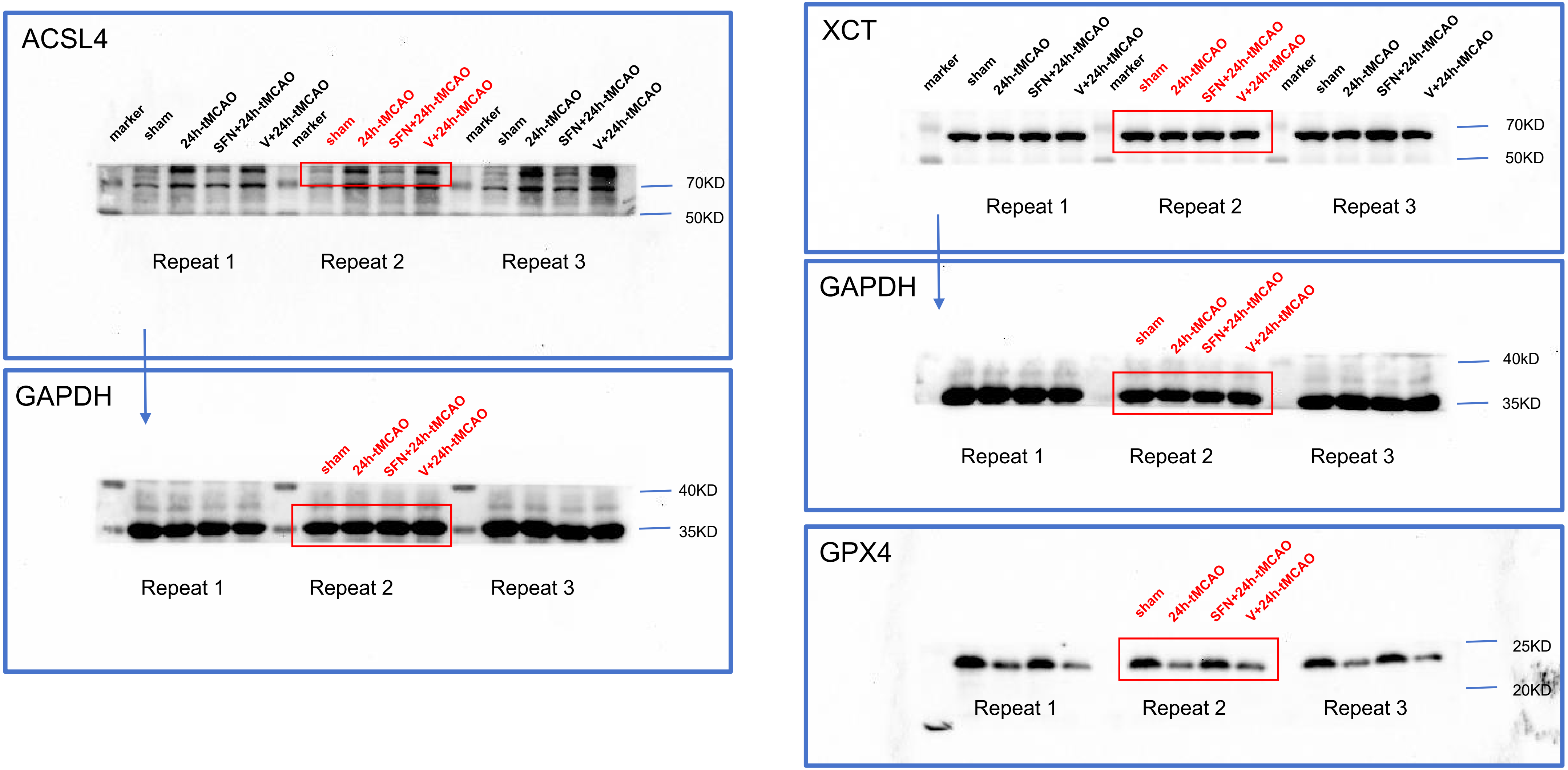

Supplementary Figure S6. Original Western blots of Figure 7C (red box circled). We completed the Western-blot experiment shown in this picture in the same gel. However, in order to incubate different primary antibodies, western blots were cropped prior to incubation with primary antibody hybridization.. We repeated the experiment three times on a single gelatin plate.

Figure S1-NRF2

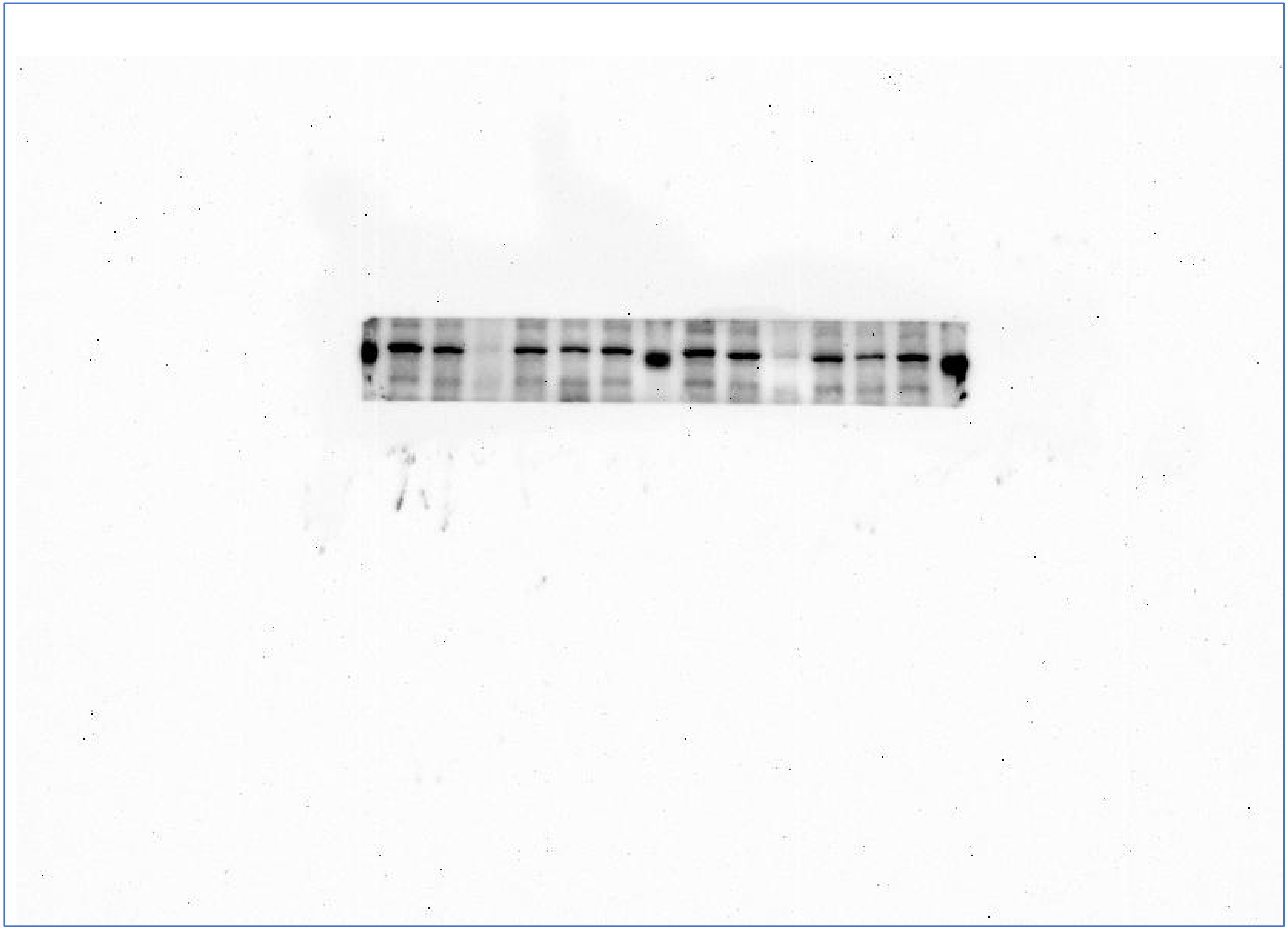

Figure S1-GAPDH

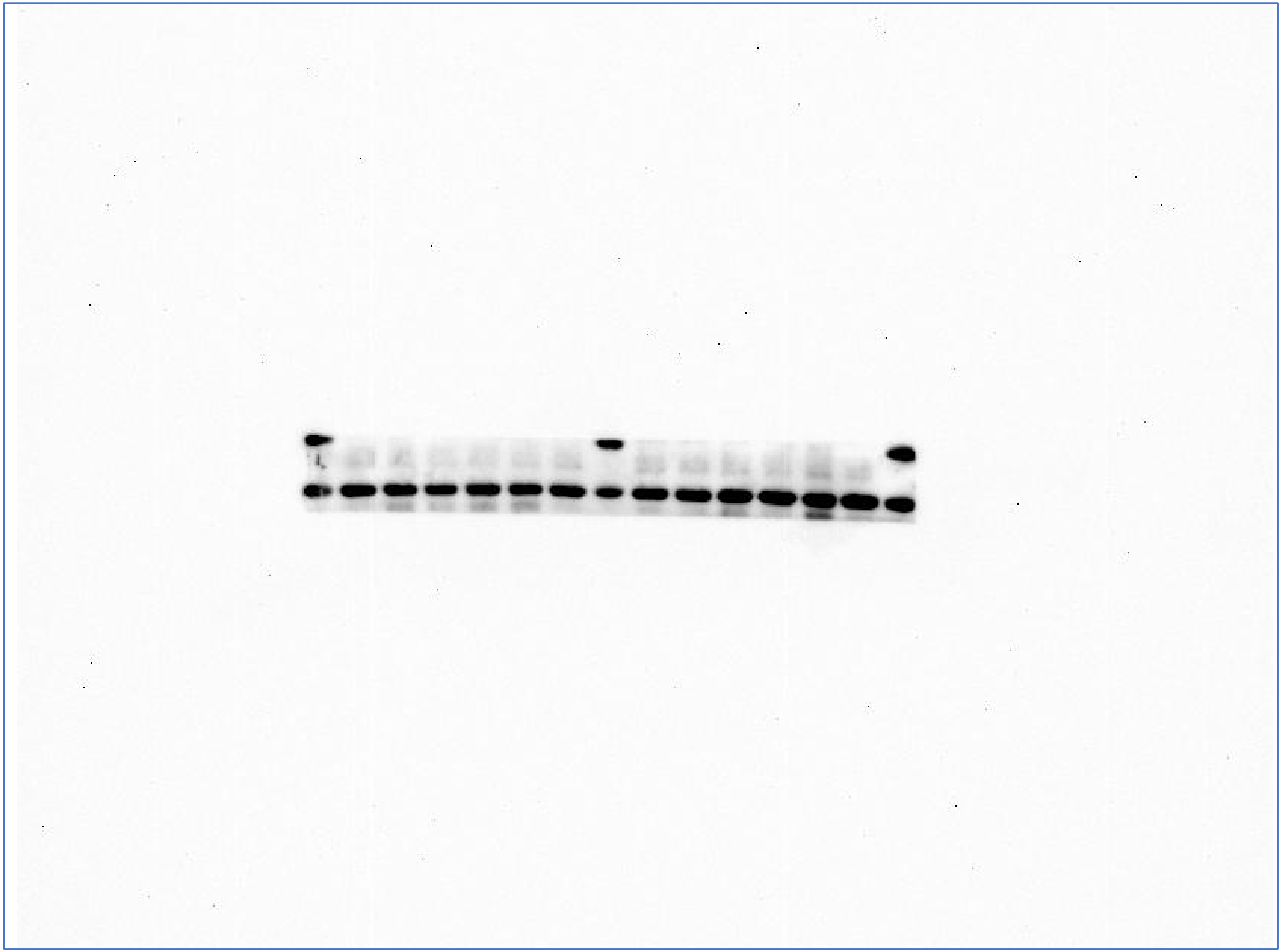

Figure S2-NRF2

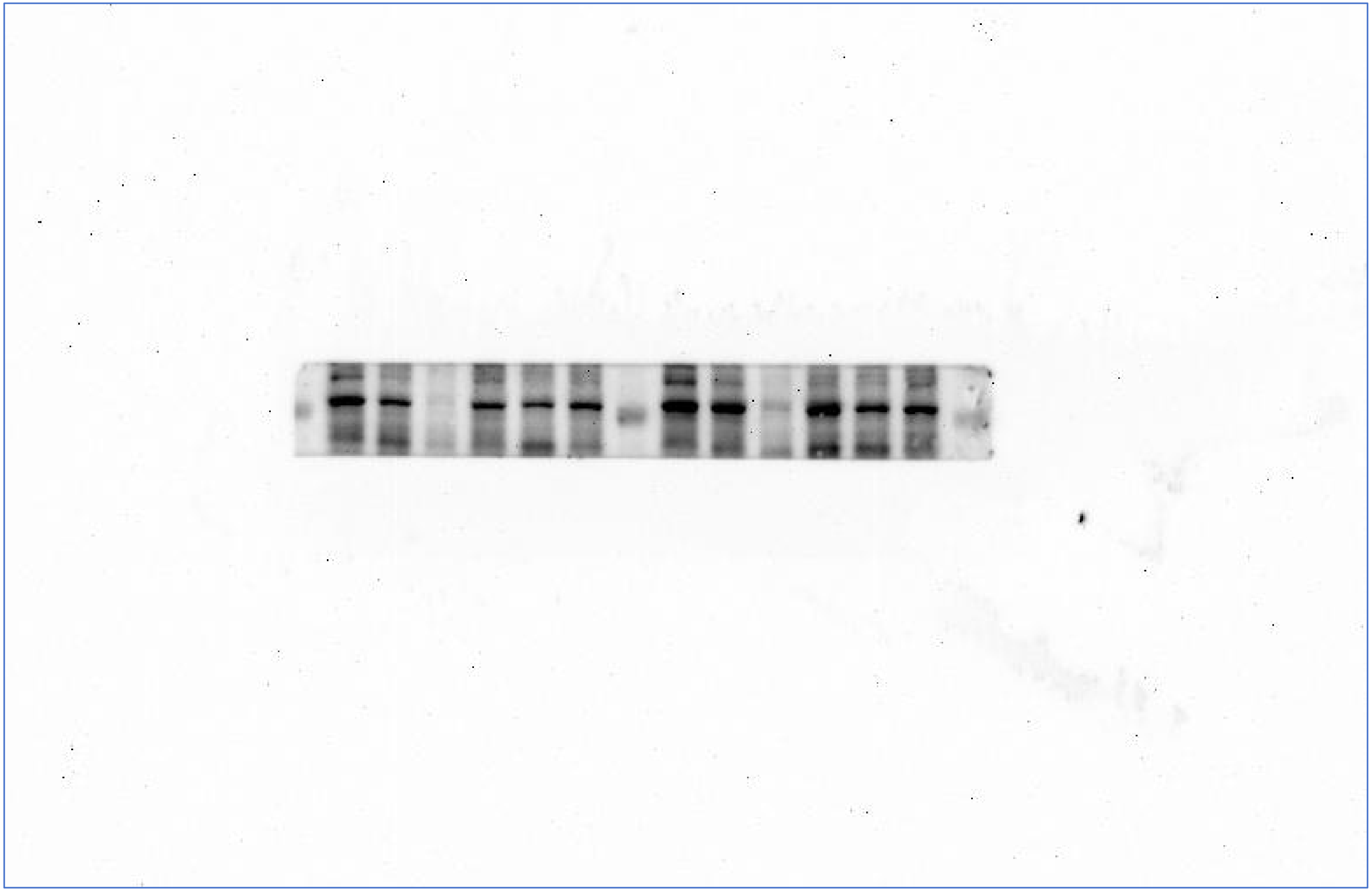

Figure S2-GAPDH

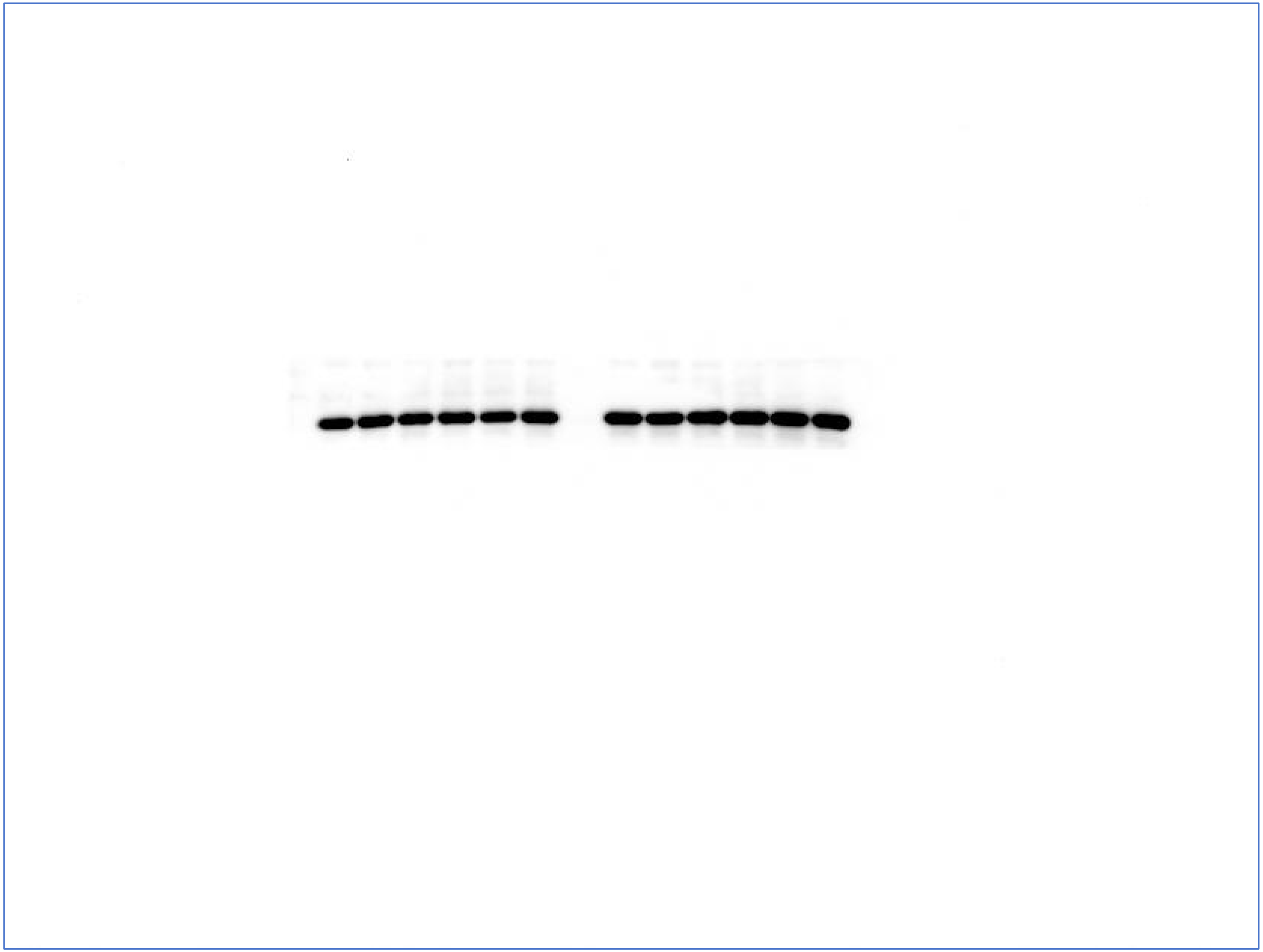

Figure S3-ZO-1

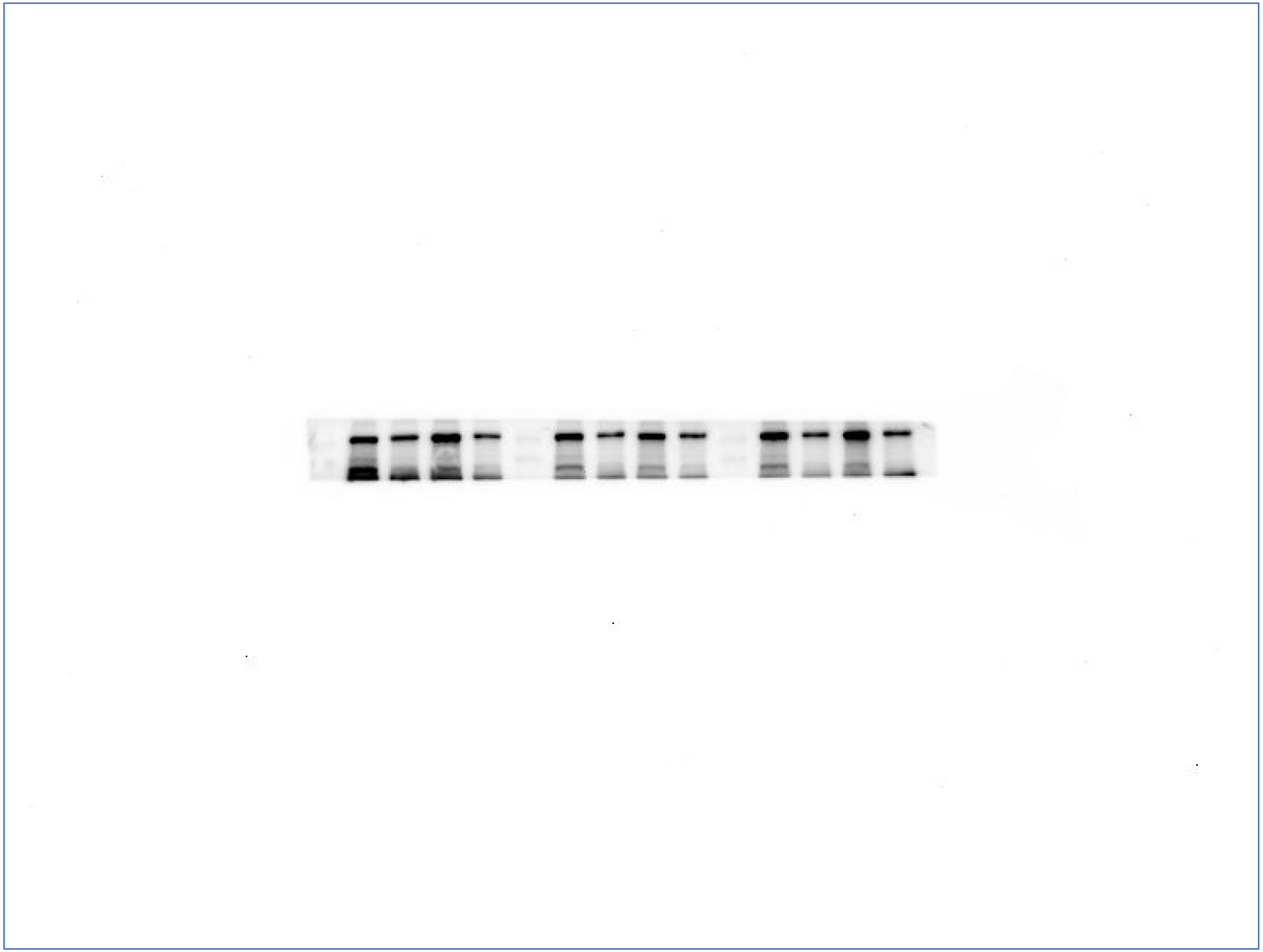

Figure S3-Occludin

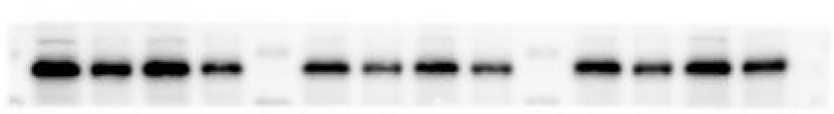

Figure S3-GAPDH

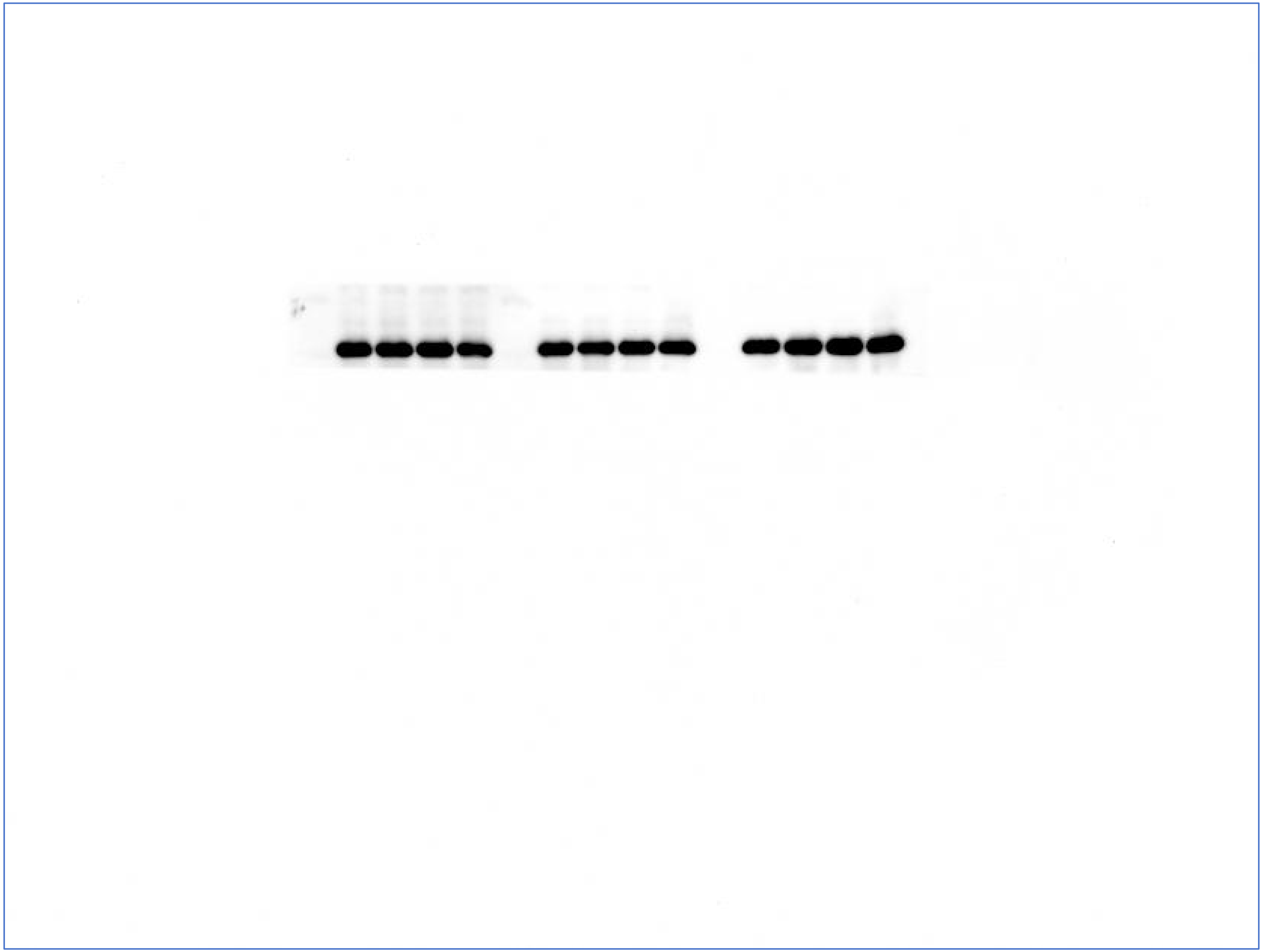

Figure S4-MMP9

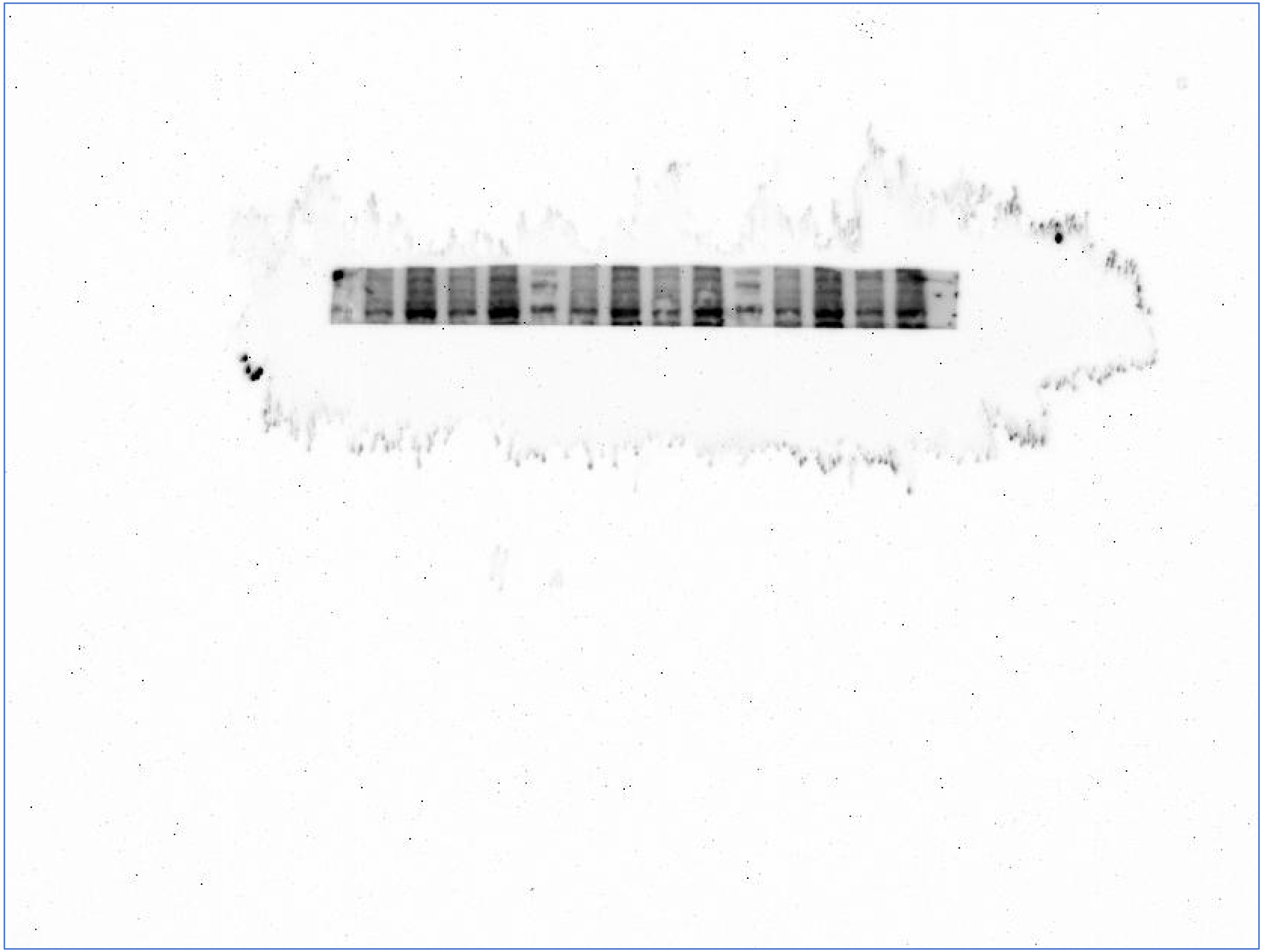

Figure S4-AQP4

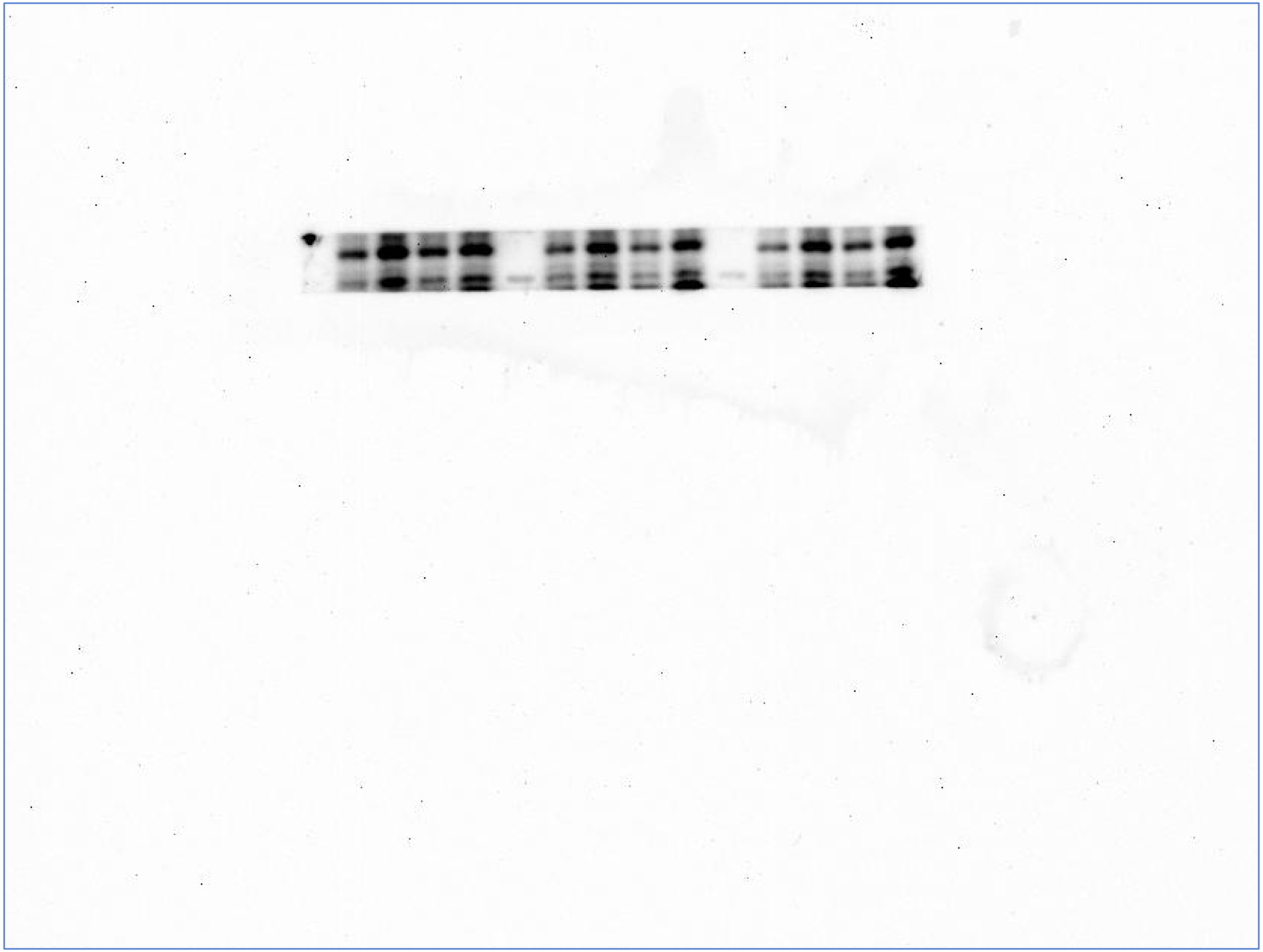

Figure S4-GAPDH

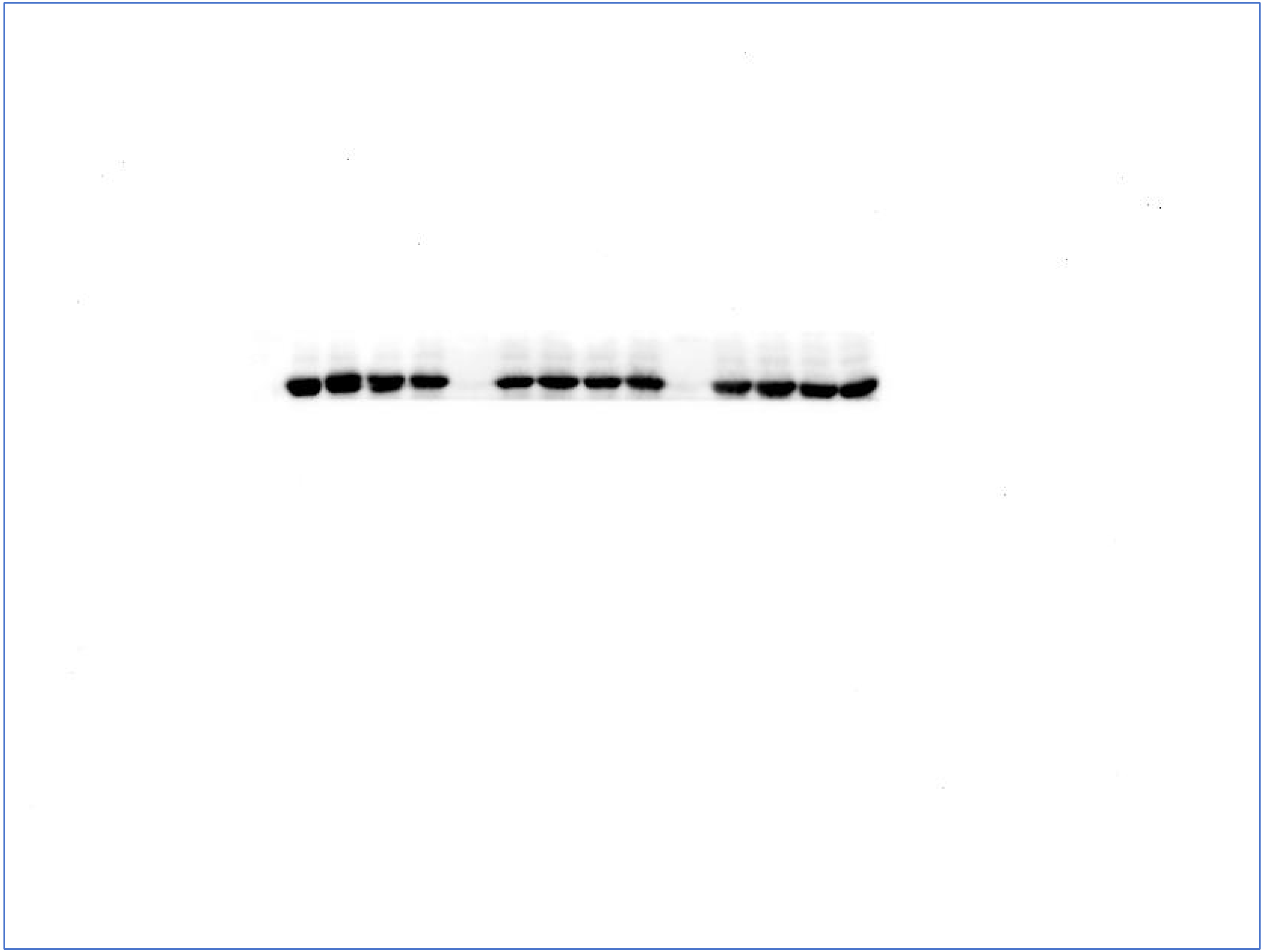

Figure S5-p-NK-κB

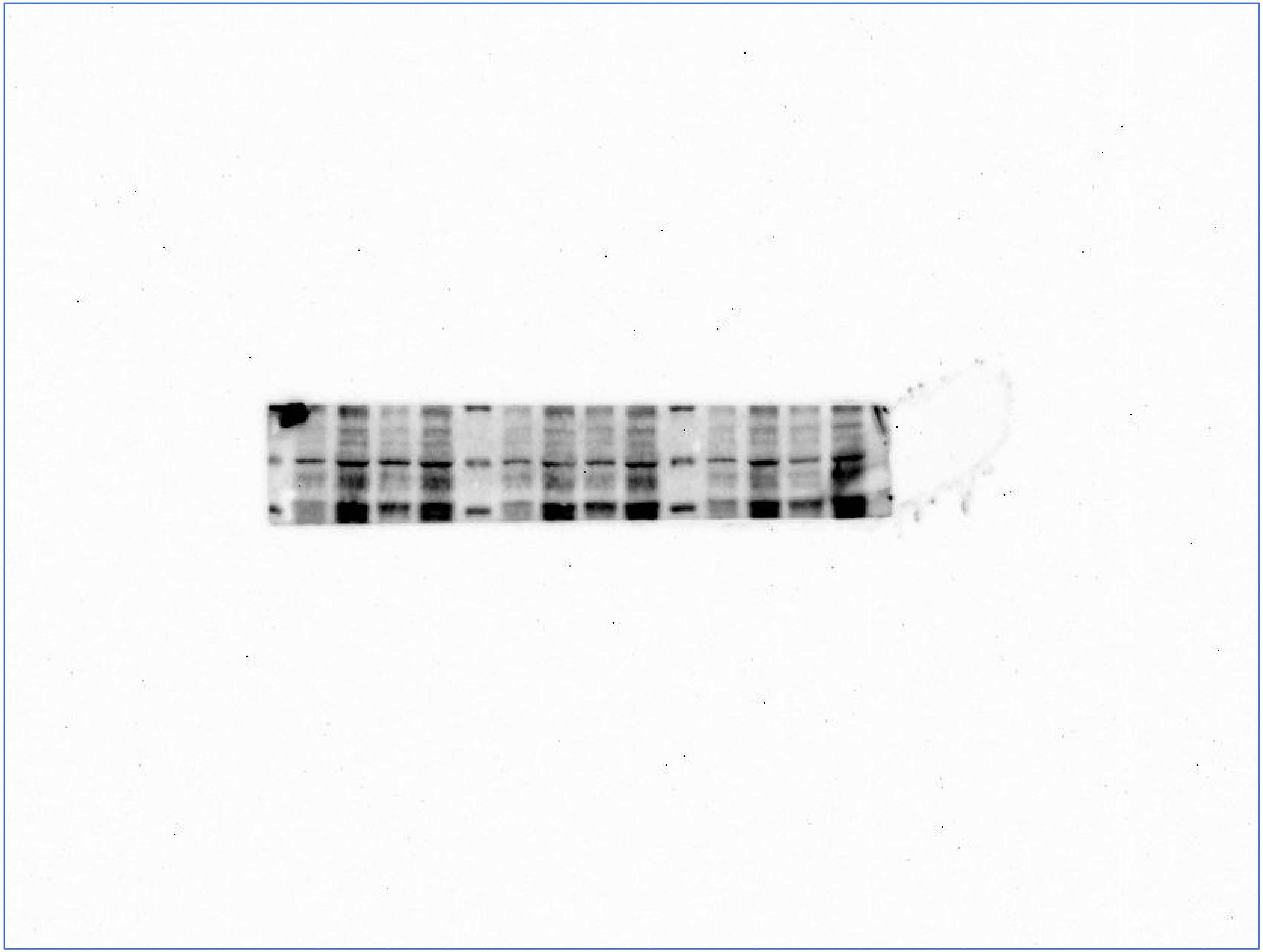

Figure S5-IL-18

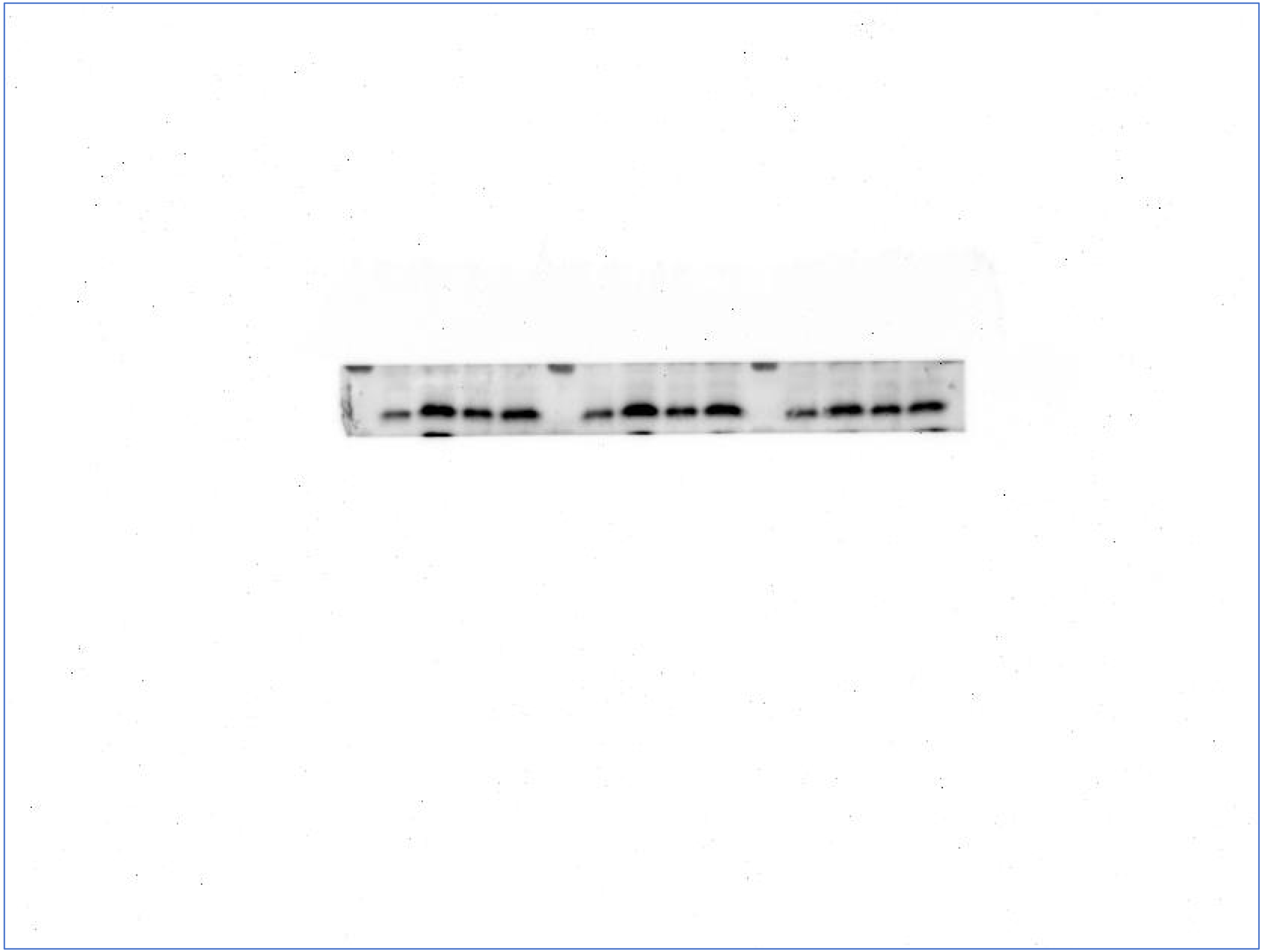

Figure S5-IL-1β

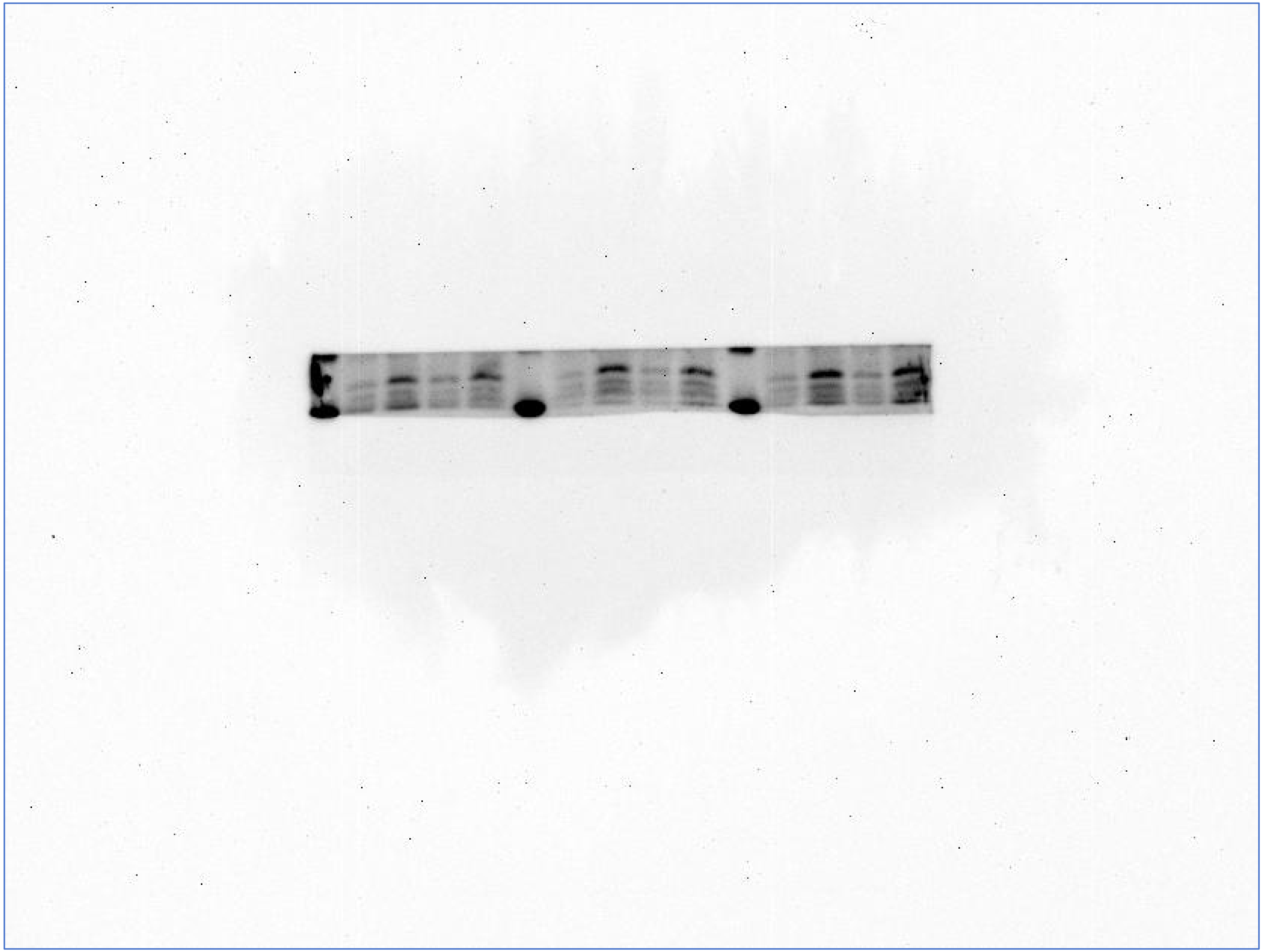

Figure S5-GAPDH

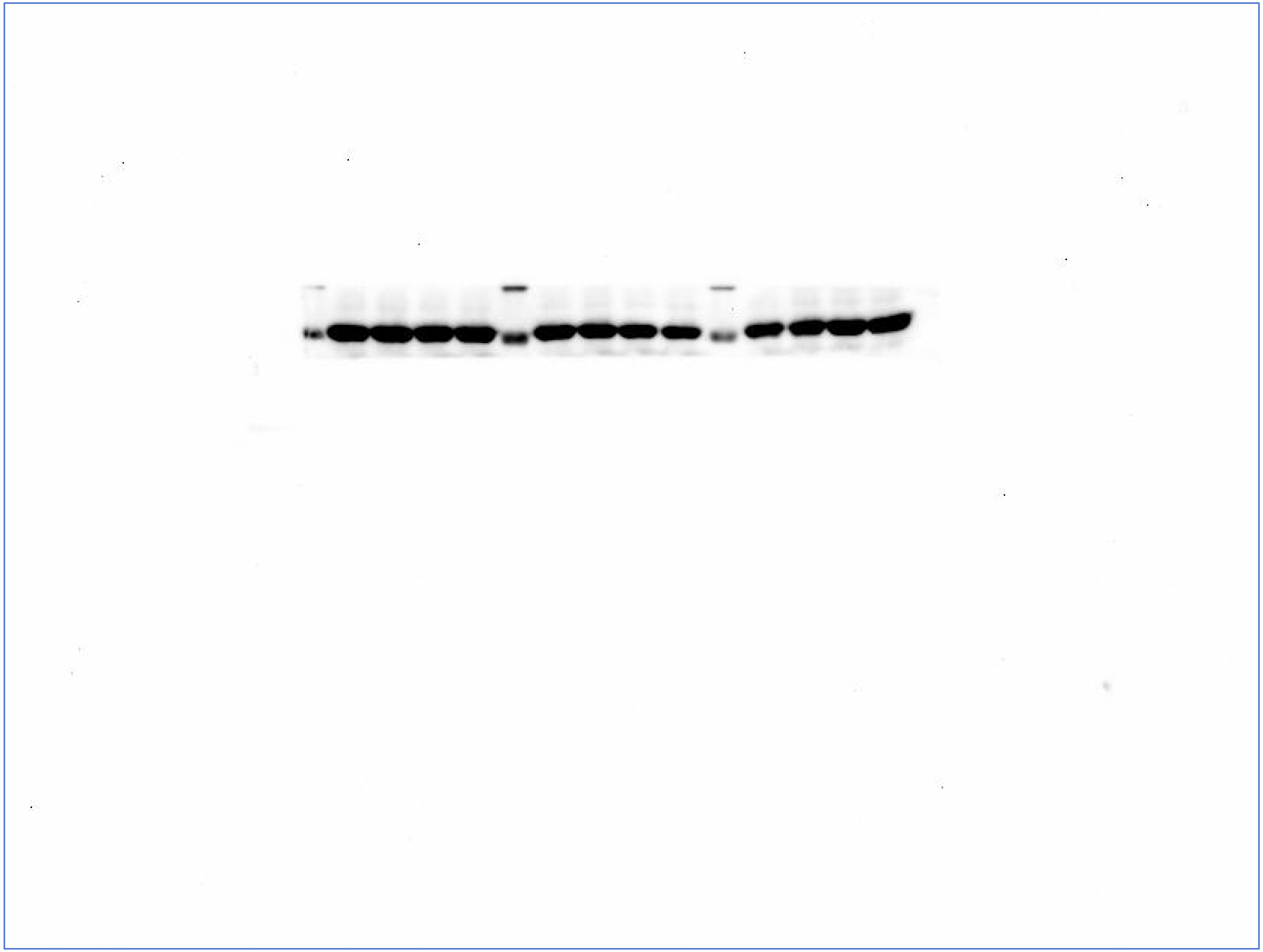

Figure S6-ACSL4

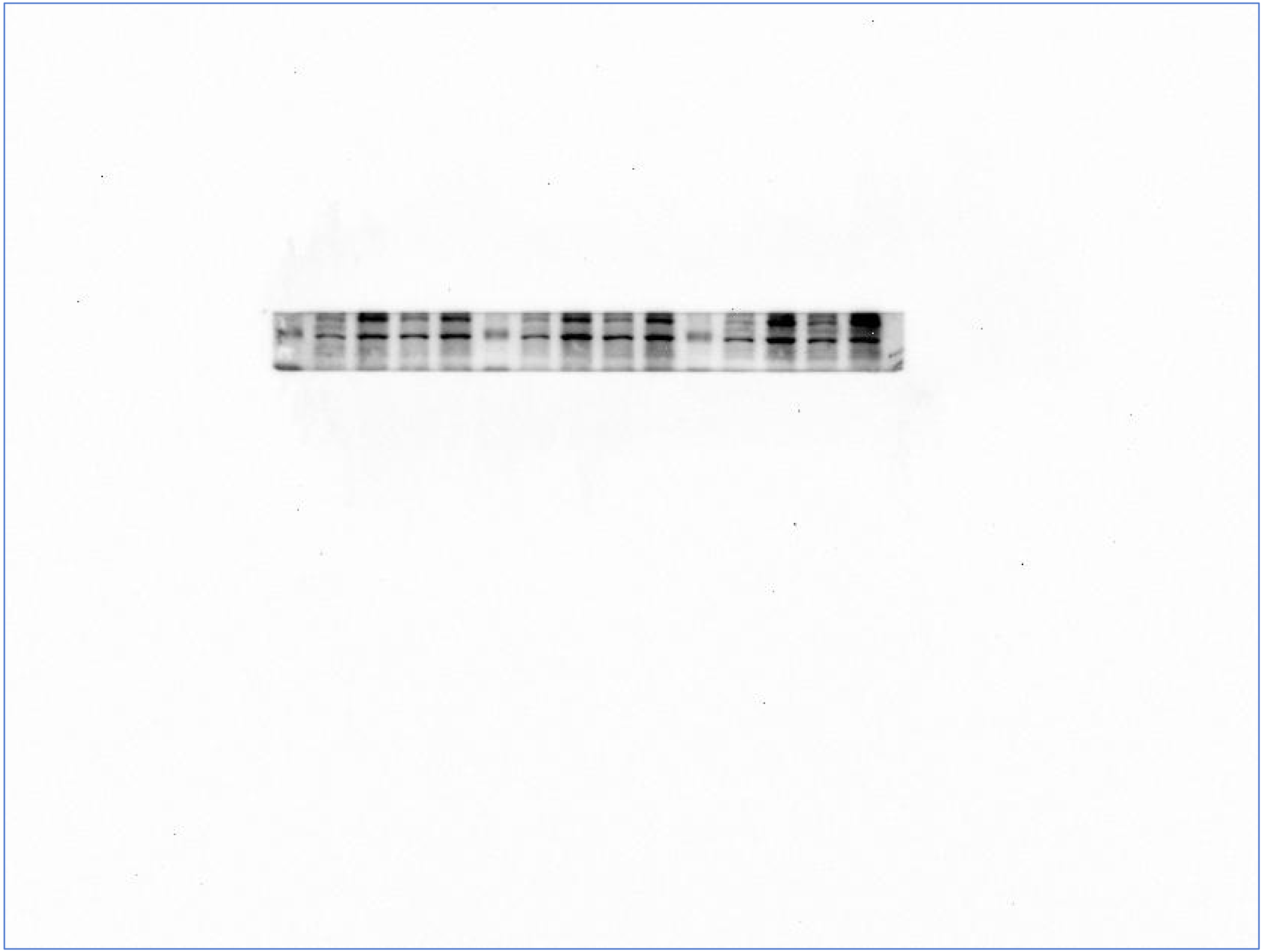

Figure S6-GAPDH

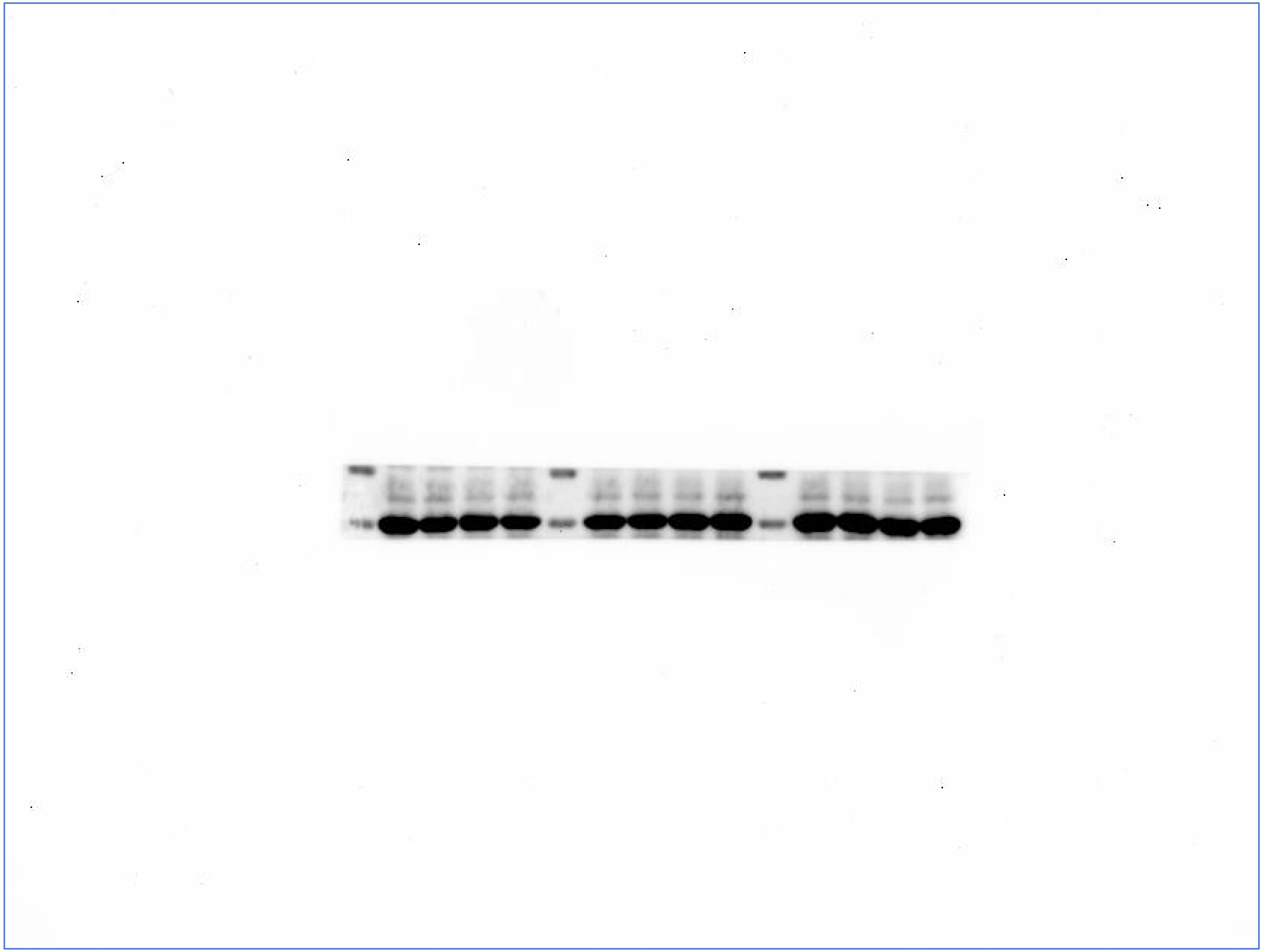

Figure S6-XCT

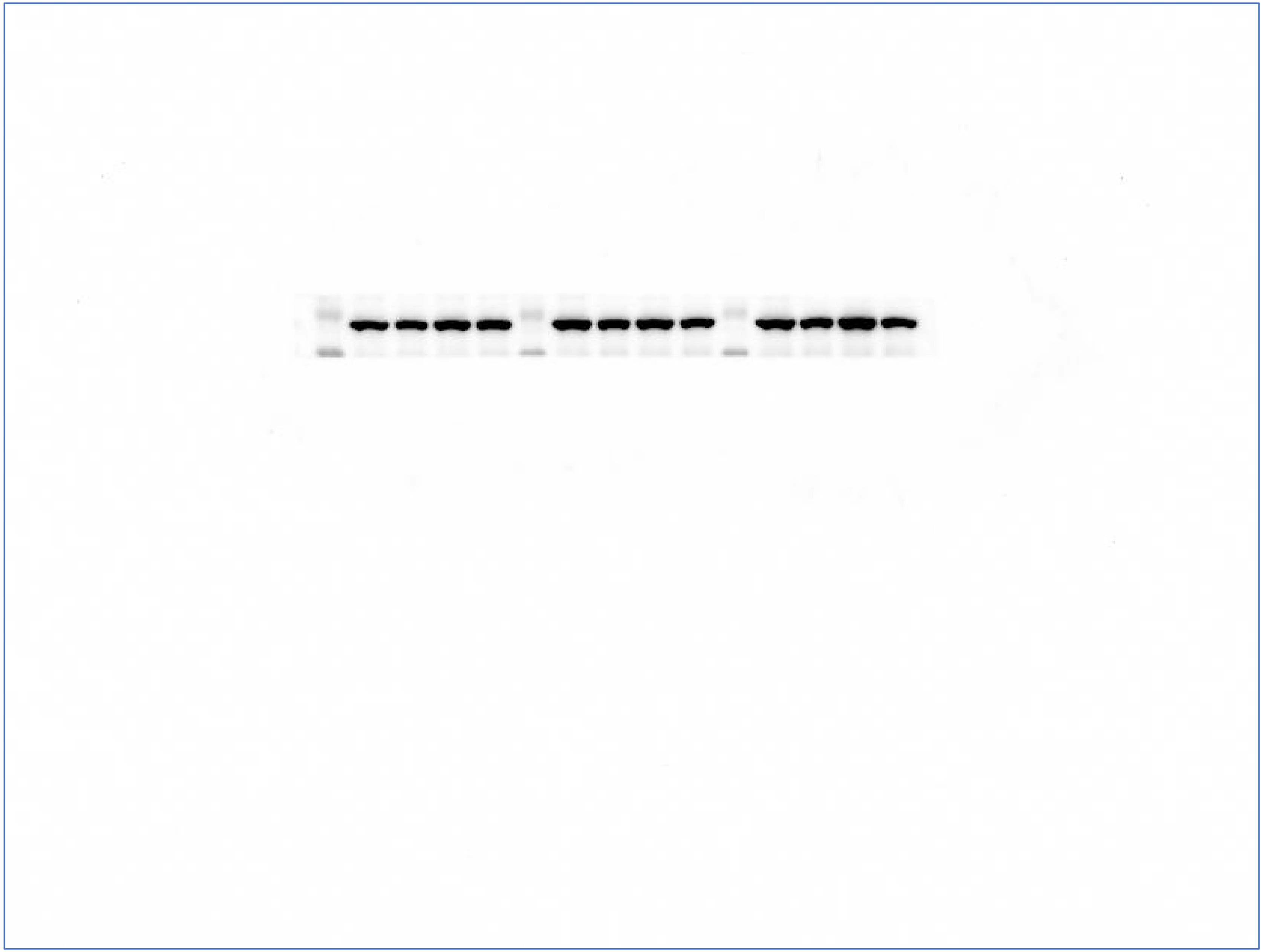

Figure S6-GPX4

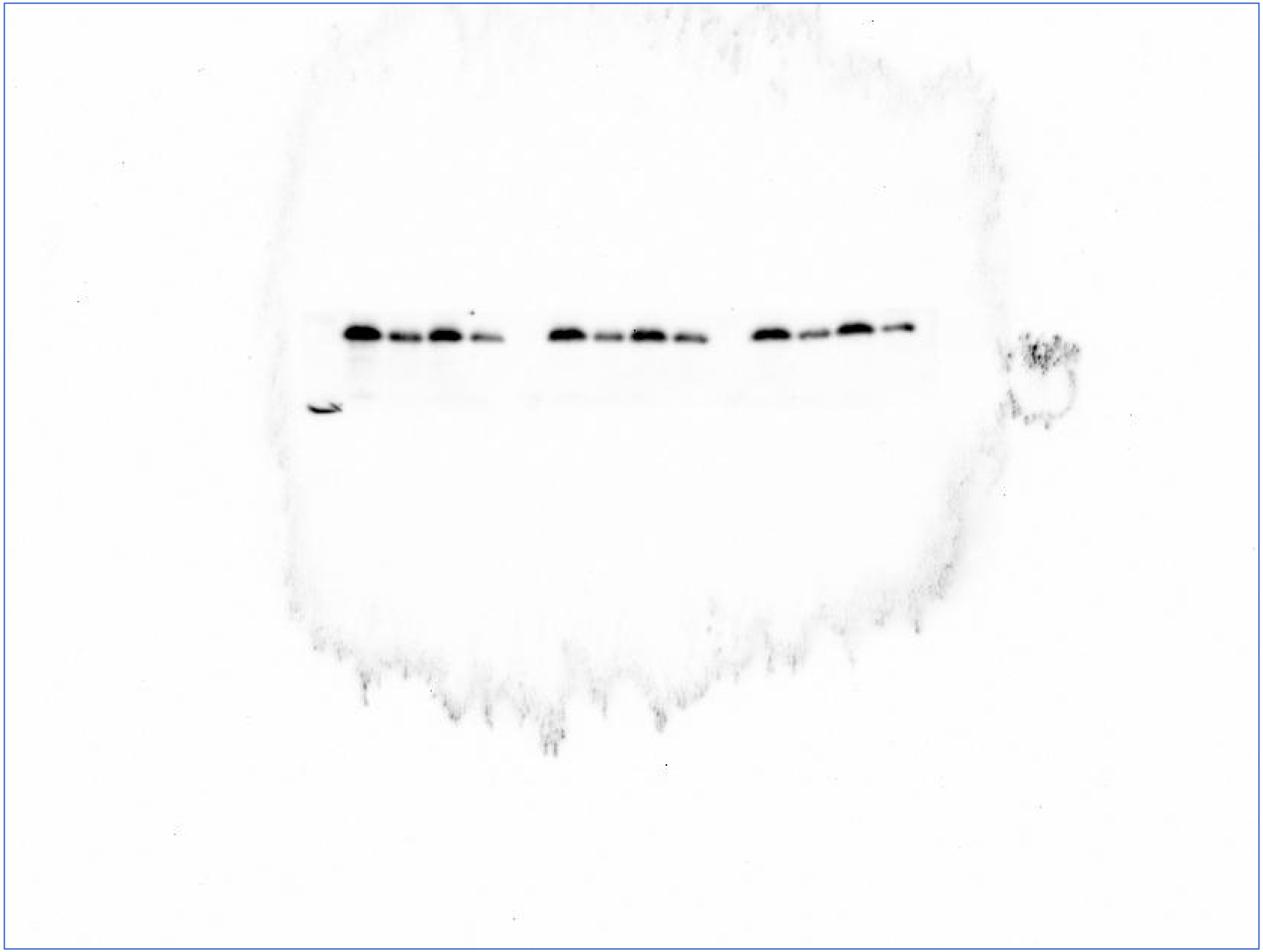

Figure S6-GAPDH

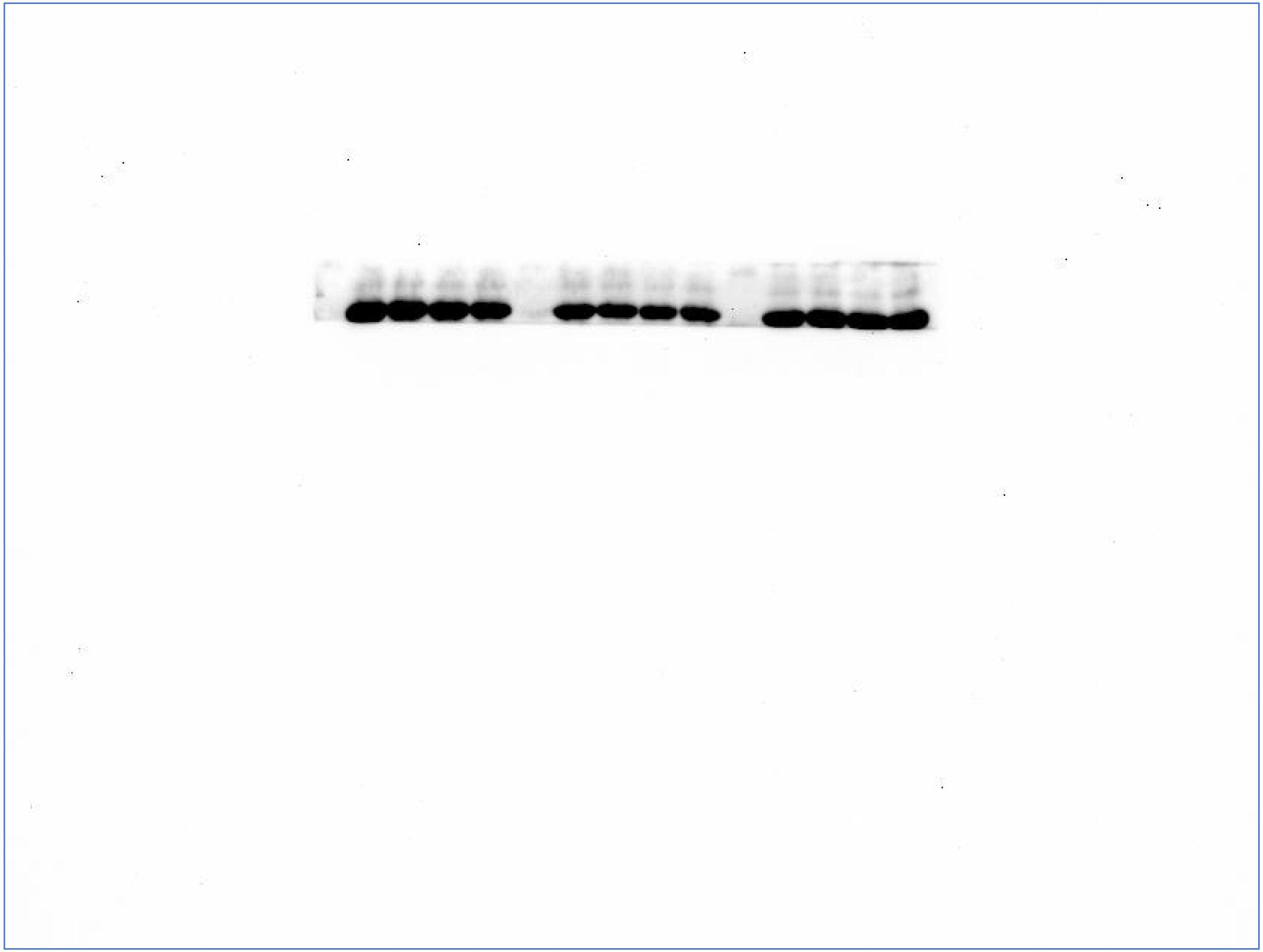

Supplement: Supplementary file 1 — Supplementary Figures. [file 41598_2024_53836_MOESM1_ESM.pdf]
